# Cyanorhodopsin-II represents a yellow-absorbing proton-pumping rhodopsin clade within cyanobacteria

**DOI:** 10.1093/ismejo/wrae175

**Published:** 2024-11-01

**Authors:** Masumi Hasegawa-Takano, Toshiaki Hosaka, Keiichi Kojima, Yosuke Nishimura, Marie Kurihara, Yu Nakajima, Yoshiko Ishizuka-Katsura, Tomomi Kimura-Someya, Mikako Shirouzu, Yuki Sudo, Susumu Yoshizawa

**Affiliations:** Atmosphere and Ocean Research Institute, The University of Tokyo, Chiba 277–8564, Japan; Graduate School of Frontier Sciences, The University of Tokyo, Chiba 277–8563, Japan; Institute for Extra-Cutting-Edge Science and Technology Avant-Garde Research (X-star), Japan Agency for Marine-Earth Science and Technology (JAMSTEC), Kanagawa 237–0061, Japan; Laboratory for Protein Functional and Structural Biology, RIKEN Center for Biosystems Dynamics Research, Kanagawa 230–0045, Japan; Faculty of Medicine, Dentistry and Pharmaceutical Sciences, Okayama University, Okayama 700–8530, Japan; Atmosphere and Ocean Research Institute, The University of Tokyo, Chiba 277–8564, Japan; Research Center for Bioscience and Nanoscience (CeBN), Research and Development Center for Marine Biosciences, Japan Agency for Marine-Earth Science and Technology (JAMSTEC), Kanagawa 237–0061, Japan; Graduate School of Medicine, Dentistry and Pharmaceutical Sciences, Okayama University, Okayama 700–8530, Japan; Atmosphere and Ocean Research Institute, The University of Tokyo, Chiba 277–8564, Japan; Institute for Extra-Cutting-Edge Science and Technology Avant-Garde Research (X-star), Japan Agency for Marine-Earth Science and Technology (JAMSTEC), Kanagawa 237–0061, Japan; Research Center for Bioscience and Nanoscience (CeBN), Research and Development Center for Marine Biosciences, Japan Agency for Marine-Earth Science and Technology (JAMSTEC), Kanagawa 237–0061, Japan; Laboratory for Protein Functional and Structural Biology, RIKEN Center for Biosystems Dynamics Research, Kanagawa 230–0045, Japan; Laboratory for Protein Functional and Structural Biology, RIKEN Center for Biosystems Dynamics Research, Kanagawa 230–0045, Japan; Laboratory for Protein Functional and Structural Biology, RIKEN Center for Biosystems Dynamics Research, Kanagawa 230–0045, Japan; Faculty of Medicine, Dentistry and Pharmaceutical Sciences, Okayama University, Okayama 700–8530, Japan; Atmosphere and Ocean Research Institute, The University of Tokyo, Chiba 277–8564, Japan; Graduate School of Frontier Sciences, The University of Tokyo, Chiba 277–8563, Japan; Collaborative Research Institute for Innovative Microbiology, The University of Tokyo, Tokyo 113–8657, Japan

**Keywords:** cyanobacteria, microbial rhodopsin, ecology, evolution

## Abstract

Microbial rhodopsins are prevalent in many cyanobacterial groups as a light-energy-harvesting system in addition to the photosynthetic system. It has been suggested that this dual system allows efficient capture of sunlight energy using complementary ranges of absorption wavelengths. However, the diversity of cyanobacterial rhodopsins, particularly in accumulated metagenomic data, remains underexplored. Here, we used a metagenomic mining approach, which led to the identification of a novel rhodopsin clade unique to cyanobacteria, cyanorhodopsin-II (CyR-II). CyR-IIs function as light-driven outward H^+^ pumps. CyR-IIs, together with previously identified cyanorhodopsins (CyRs) and cyanobacterial halorhodopsins (CyHRs), constitute cyanobacterial ion-pumping rhodopsins (CyipRs), a phylogenetically distinct family of rhodopsins. The CyR-II clade is further divided into two subclades, YCyR-II and GCyR-II, based on their specific absorption wavelength. YCyR-II absorbed yellow light (λ_max_ = 570 nm), whereas GCyR-II absorbed green light (λ_max_ = 550 nm). X-ray crystallography and mutational analysis revealed that the difference in absorption wavelengths is attributable to slight changes in the side chain structure near the retinal chromophore. The evolutionary trajectory of cyanobacterial rhodopsins suggests that the function and light-absorbing range of these rhodopsins have been adapted to a wide range of habitats with variable light and environmental conditions. Collectively, these findings shed light on the importance of rhodopsins in the evolution and environmental adaptation of cyanobacteria.

## Introduction

Almost all biological processes on Earth are driven by the energy supplied by sunlight. Chlorophyll-based photosystems convert sunlight energy into chemical energy through photosynthetic reactions, and this energy flows into ecosystems in the form of organic compounds. Cyanobacteria, one of the most abundant photosynthetic microorganisms utilizing chlorophyll-based photosystems, inhabit almost any light-exposed environment [1, 2]. This boundless environmental adaptation is supported by light harvesting through their highly diverse chlorophyll-based photosystems. For example, cyanobacterial lineages have differentiated absorptive chlorophyll pigments present in photosystem I and II reaction centers [3–6]. In addition, various lineages employ a light-harvesting antenna known as the phycobilisome. The phycobilisome is efficiently used by various lineages to capture available wavelength in the environment, such as marine *Synechococcus* strains that use phycobilisome to capture blue-to-green light available in their habitats [7, 8].

Microbial rhodopsins, another light-utilizing system, are distributed in broad bacterial taxa, including cyanobacteria [9–13]. Rhodopsin is a photoreceptor membrane protein that binds retinal as the sole chromophore, a simpler light-utilizing system than the chlorophyll-based photosystem, which consists of many proteins and typically multiple chromophores. All rhodopsins are activated by visible light and return to their original state through a photocycle that forms various intermediates (called photo-intermediates) [14]. During the photocycle, they exhibit their cognate protein functions such as ion transport [15]. In contrast, absorption wavelengths of the retinal chromophore are not homogeneous. For example, bacteriorhodopsins (BRs) of halophilic archaea, light-driven outward H^+^ pumping rhodopsins, absorb yellow light (λ_max_ = 570 nm) [16]. Proteorhodopsins (PRs) of marine bacteria are light-driven outward H^+^ pumping rhodopsins that are divided into two types of absorption maxima: 490 nm (blue-absorbing PR, BPR) [17] and 500–525 nm (green-absorbing PR, GPR) [18]. The former is relatively abundant in blue-light enriched deep/pelagic waters and the latter green-light-enriched surface/coastal waters [19, 20]. The difference in absorption maxima is thought to be the result of adaptation to the light conditions of their habitats.

Two cyanobacteria-specific rhodopsin clades, namely cyanobacterial halorhodopsin (CyHR) and cyanorhodopsin (CyR), have been identified as light-driven inward Cl^−^ and outward H^+^ pumping rhodopsin clades, respectively [21]. The discovery of these rhodopsin clades unique to cyanobacteria suggests that the rhodopsin-mediated and chlorophyll-based photosystems have long coexisted in cyanobacterial cells. These photosystems have complementary absorption wavelength ranges, allowing for harmonized capture of sunlight energy in cyanobacterial cells [21]. However, due to the limited rhodopsin explorations focusing on cultured strains [9, 11–13, 21], the distribution of rhodopsin genes within cyanobacterial lineages and the phylogenetic diversity of cyanobacterial rhodopsins remain underexplored, considering the accumulated metagenomic data such as numerous metagenome-assembled genomes (MAGs) [22].

In this study, we explored rhodopsin sequences in both cyanobacterial genomes and metagenomic assemblies. Phylogenetic analysis of the detected rhodopsin sequences led to the identification of three cyanobacteria-specific rhodopsin clades, including a new clade Cyanorhodopsin-II (CyR-II). CyR-II was characterized with the predominance of uncultured cyanobacteria. Spectroscopic, X-ray structural, and mutational analyses demonstrate that the rhodopsins in the clade found in this study have red-shifted absorption maxima due to slight changes in the side-chain structure.

## Materials and methods

Rhodopsin genes of cyanobacteria were identified as follows. Cyanobacterial genome assemblies and their coding sequences were downloaded from the National Center for Biotechnology Information (NCBI) and the US Department of Energy Joint Genome Institute (JGI) Integrated Microbial Genomes (IMG) database. In addition, the OceanDNA MAG catalog [22] and metagenomic assemblies derived from metagenomes published in previous studies [23–26] were also used to explore the rhodopsin genes. Genes encoding rhodopsins were explored using two hidden Markov models (Supporting Data S1 and S2) [27] constructed from large-scale sequence alignments of known rhodopsins and their homologs. The phylogenetic trees of rhodopsins and cyanobacterial genomes were reconstructed using IQ-TREE (v. 1.6.12) [28] by 1000 ultrafast bootstrap searches [29]. Habitat and morphological information was collected manually: habitat information is based on “isolation source” of genome assembly in NCBI or the previous report [30] and morphological classifications are based on the previous report [31]. Data files of sequence alignments and phylogenies of rhodopsin and cyanobacterial genomes are available (Supporting Data S3–S8).

The codon-optimized DNA fragments to *Escherichia coli* encoding the P7104R (*Nodosilinea nodulosa* PCC 7104 rhodopsin, Accession No. WP_017301364.1), CBR35R (*Chroococcidiopsidaceae* cyanobacterium CP_BM_RX_35, MBV8882851.1), and MAG18R (uncultured cyanobacterium SRR6869040_bin.18, SRR6869040_N0001714_12) genes were chemically synthesized and inserted into the pET21a (+) plasmid vector by Eurofins Genomics (Japan). The ion transport activities of the CyR-IIs were examined by monitoring the light-induced pH changes in suspensions of CyR-IIs-expression *E. coli* cells. Ion transport measurements were repeated under the same conditions after the addition of the proton-selective ionophore, 30 μM carbonyl cyanide *m*-chlorophenylhydrazone (CCCP; Sigma-Aldrich, USA).

For spectroscopic analyses, the His-tagged CyR-II proteins (P7104R, CBR35R, and MAG18R) were expressed in *E. coli* C41 (DE3) (Lucigen, USA). Cells were solubilized with 1.0% (w/v) *n*-dodecyl-β-d-maltoside (DDM, Dojindo Lab., Japan). The solubilized-fraction was purified by Ni^2+^ affinity column chromatography. All UV–Vis spectra were measured using a UV-2450 spectrophotometer (Shimadzu, Japan).

The retinal isomer composition was determined by using high-performance liquid chromatography as previously described [21, 32]. The pH of the purified P7104R was adjusted to the desired values (1.11–11.17) to ensure that no denaturation occurred. The absorption spectrum (250–750 nm) was measured for samples under the various pH conditions. The p*K*_a_ value or values were then estimated by fitting the data to the Henderson–Hasselbalch equation with two (p*K*_a1_ and p*K*_a2_) or one p*K*_a_.

For the flash-photolysis experiment, crude membranes of P7104R were obtained by disruption by sonication on ice-cold water and ultracentrifugation. Time-resolved absorption spectra from 370 to 720 nm at 5-nm intervals in purified P7104R and from 390 to 720 nm at 10-nm intervals in *E. coli* membrane expressing P7104R were measured by using a computer-controlled flash-photolysis system. The wavelength of the actinic light pulse was tuned to 565 nm. The data at 565 and 570 nm in purified P7104R were excluded from the analysis to account for the scattering of the light pulses. To monitor proton uptake and release during the photocycle, we repeated the flash-photolysis experiment using purified P7104R under the same conditions after the addition of the pH indicator pyranine (final concentration = 100 μM; Tokyo Chemical Industry Co., Ltd., Japan), which is a pH-sensitive fluorochrome.

The P7104R protein used for crystallization was synthesized by using an *E. coli* cell-free protein synthesis system according to previously reported protocols [33–36] and crystallized by the *in meso* method. Diffraction data for P7104R were collected at the BL32XU beamline of the SPring-8 synchrotron by using the multiple small-wedge scheme implemented in the ZOO system [37, 38]. Data processing was performed using the KAMO program [39], and the structure was solved by molecular replacement using the Phaser program [40] in the Phenix suite [41]. The search model was the BR [42], and the structure was manually rebuilt with the Coot program [43].

## Results

### Collection of cyanobacterial rhodopsins and their phylogeny

**Figure 1:**
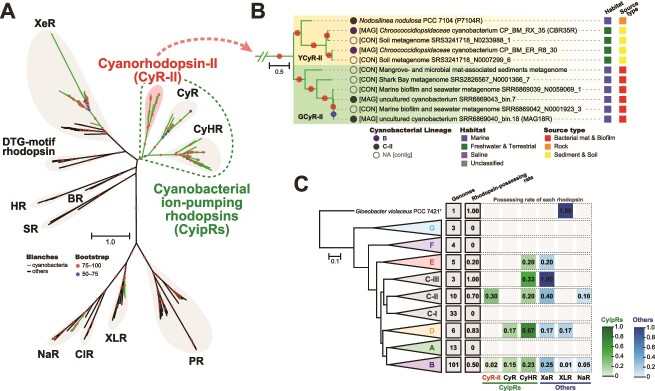
Phylogeny of cyanobacterial rhodopsins and distribution among cyanobacterial lineages: (A) A maximum likelihood tree of amino acid sequences of 102 cyanobacterial and 69 non-cyanobacterial rhodopsins; rhodopsin clades are indicated as follows: NaR, ClR (Cl^−^ pumping rhodopsin), XLR, PR, SR (sensory rhodopsin I and sensory rhodopsin II), BR, HR (halorhodopsin), DTG-motif rhodopsin, XeR, CyHR, CyR, and an uncharacterized cyanobacteria-specific clade (CyR-II); CyipRs are phylogenetically distinct cyanobacteria-specific rhodopsins consisting of CyR-II, CyR, and CyHR; the green branches indicate cyanobacterial rhodopsins, and the black branches indicate others; bootstrap probabilities (≥50%) are indicated by colored circles; the scale bar represents the number of substitutions per site; (B) An enlarged view of the novel cyanobacteria-specific clade CyR-II with habitat and source type information; the clade is divided into two subclades: YCyR-II (yellow-absorbing CyR-II) and GCyR-II (green-absorbing CyR-II); the circles in front of the sequence names were colored based on cyanobacterial lineages; names starting with [CON] and [MAG] indicate that the sequence is from the metagenomic contig and MAG, respectively; (C) distribution of rhodopsins in cyanobacterial lineages; the phylogenomic tree was constructed by maximum likelihood estimation based on conserved amino acid sites of 120 ubiquitous single-copy genes of bacteria and collapsed by lineages; “genomes” indicates the number of genomes per lineage, and “rhodopsin-harboring genomes” indicates the number of genomes containing rhodopsin; the numbers within the dashed squares indicate the fraction of genomes that contain each clade of rhodopsins in comparison with the total number of genomes in a lineage.

We explored rhodopsin genes through a homology search against cyanobacterial genomes in databases and cyanobacteria-affiliated contigs assembled from publicly available metagenomic data. A total of 102 rhodopsin genes were identified, 96 of which were from genomes and 6 from metagenomic contigs. Phylogenetic analysis was performed using the 102 cyanobacterial rhodopsins in addition to noncyanobacterial rhodopsins. The 91 rhodopsins were classified into five previously reported clades, including those derived from cyanobacteria: xanthorhodopsin-like rhodopsin (XLR, 3 genes), Na^+^ pumping rhodopsin (NaR, 6 genes), xenorhodopsin (XeR, 35 genes), CyHR (31 genes), and CyR (16 genes). The remaining 11 rhodopsins constitute a new rhodopsin clade with high bootstrap support (100%) (Figs 1A and S1). We name the clade CyR-II, which contains 10 metagenome-derived rhodopsins (four from MAGs and six from metagenomic contigs), and only one rhodopsin derived from an isolated strain (*Nodosilinea nodulosa* PCC 7104). The CyR-II clade was divided into two subclades, YCyR-II and GCyR-II, with high bootstrap support (99% for both). These two subclades have characteristic environmental distributions: YCyR-II mainly from sediment and soil of terrestrial environments, and GCyR-II from bacterial mats and biofilms of marine environments (Fig. 1B). The three clades (CyHR, CyR, and CyR-II) collectively constitute a phylogenetically distinct family of cyanobacteria-specific rhodopsins, which is designated as “cyanobacterial ion-pumping rhodopsins” (CyipRs) in this study.

To investigate the evolutionary process of rhodopsin acquisition and deletion in cyanobacterial genomes, we performed a phylogenomic reconstruction of cyanobacteria. A phylogenomic tree based on 120 single-copy marker genes confirmed that cyanobacteria are divided into seven lineages (A–G) that are referred to as subclades in previous studies [21, 44], with the slight difference that the lineage of *Leptolyngbya ohadii* IS1 is shifted from C to D (Figs 1C and S2). Lineage C was further divided into three lineages with high bootstrap support (C-I–III). *Gloeobacter violaceus* PCC 7421^T^, thought to be the earliest branching cyanobacterium containing an XLR [45], does not belong to any lineage. No rhodopsin is detected in genomes of lineages A, F, and G, whereas genomes of lineages B, C-I, and D contains various clades of rhodopsins. Genomes containing CyR-II belong to lineages B (two genomes) and C-II (three genomes).

### Sequence and functional characterization of CyR-IIs

**Figure 2:**
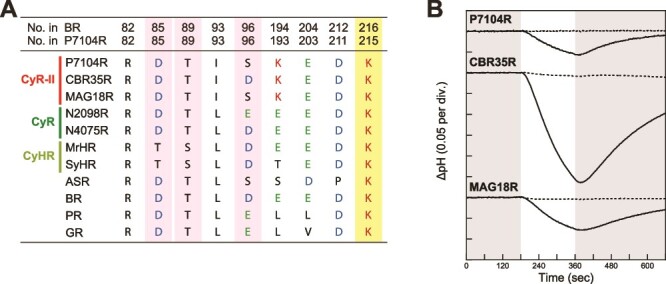
Characterization of P7104R. (A) Alignment of critical amino acids of light-driven outward H^+^ pumping rhodopsins and cyanobacterial rhodopsins. Rhodopsin names and clades (also see Fig. 1A) are indicated as follows: P7104R (*Nodosilinea nodulosa* PCC 7104 rhodopsin, CyR-II), CBR35R (*Chroococcidiopsidaceae* cyanobacterium CP_BM_RX_35 rhodopsin, CyR-II), MAG18R (uncultured cyanobacterium SRR6869040_bin.18 rhodopsin, CyR-II), N2098R (*Calothrix* sp. NIES-2098 rhodopsin, CyR), N4075R (*Tolypothrix* sp. NIES-4075 rhodopsin, CyR), MrHR (*Mastigocladopsis repens* halorhodopsin, CyHR), SyHR (*Synechocystis* halorhodopsin, CyHR), ASR (*Anabaena* sensory rhodopsin, XeR), BR (bacteriorhodopsin, BR), PR (proteorhodopsin, PR), and GR (*Gloeobacter* rhodopsin, XLR). The “No.” indicates the number of amino acids counted from the first amino acid (“start codon,” also see Fig. S3). The known function of displayed amino acids are as follows: primary proton acceptor (Asp85^BR^), proton donor (Asp96^BR^), proton release group (Glu194^BR^ and Glu204^BR^), counterion (Asp212^BR^), and Schiff base (Lys216^BR^). Two carboxylates, Asp (D) and Glu (E), are shown in blue and green, respectively, and Schiff base Lys (K) is shown in red. Specific amino acid residues (motif sequences) crucial for ion transport activity were shown by pink background (No. 85, 89, and 96 in P7104R). The Lys residue (Lys216^BR^), which forms a Schiff base linkage with the retinal, was highlighted by yellow background. (B) Light-induced changes of the pH of suspensions of *E. coli* expressing P7104R, CBR35R, and MAG18R. The changes in pH in the absence (solid line) and presence (broken line) of the proton-selective ionophore, 30 μM CCCP, are shown. The measurements were performed under the dark conditions (gray shading) with illumination at 520 ± 10 nm for 3 min (white shading, from 180 sec to 300 sec).

Specific amino acid residues (motif sequences) crucial for ion transport activity were compared after all CyR-IIs with known rhodopsins (Fig. 2A pink background and Fig. S3 for representative protein sequences). CyR-IIs have two motif types in the third helix (helix C), Asp-Thr-Ser (DTS motif, seven genes, P7104R and MAG18R in Fig. 2A) and Asp-Thr-Asp (DTD motif, four genes, CBR35R in Fig. 2A). These correspond to the Asp85, Thr89, and Asp96 (DTD motif) of BR. This indicates that CyR-IIs possess a putative proton acceptor, Asp residue (corresponding to Asp85^BR^). However, the amino acid residue of the putative proton donor, Asp or Glu residue (Asp96^BR^), differed between the two motif types. Rhodopsin binds to retinal chromophore by forming a Schiff base linkage through the Lys residue (corresponding to Lys216^BR^) in the seventh helix (helix G), which is stabilized by a negatively charged counterions (corresponding to Asp85^BR^ and Asp212^BR^). Because the Lys residue (such as Lys215^P7104R^, italic bold in Fig. 2A) in the helix G and two putative counterions (such as Asp85^P7104R^ and Asp 211^P7104R^) were conserved in all sequences other than SRS3241718_N0233988_1 (see Figs 1B and S1); almost all CyR-IIs probably can make a stable Schiff base linkage between the rhodopsin protein and the retinal chromophore and can transport protons.

To validate the prediction that CyR-IIs function as H^+^ pumps, three rhodopsin genes (P7104R, CBR35R, and MAG18R genes) were used for heterologous expression analysis. A light-induced pH decrease was observed in the suspension of the *E. coli* cells expressing each CyR-II (Fig. 2B, solid line). These pH changes were almost completely abolished in the presence of the protonophore CCCP (Fig. 2B, broken line), which collapses the proton motive force across the membrane. These results suggested that CyR-IIs export protons from the cytoplasmic side across the cytoplasmic membrane.

### Spectroscopic characterization of P7104R

**Figure 3:**
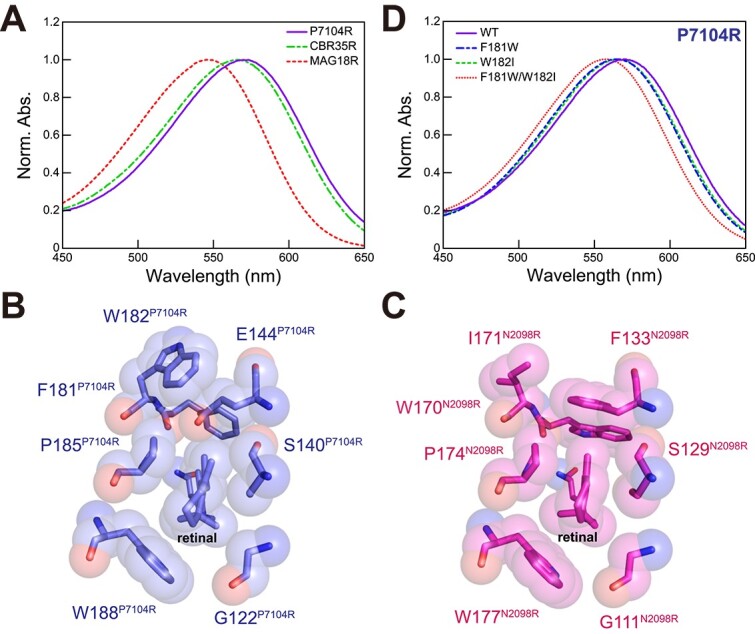
Absorption spectra of CyR-IIs and structure of P7104R. (A) UV–Vis spectra of three CyR-IIs, P7104R (purple line), CBR35R (green line), and MAG18R (red line). The location of amino acids around the retinal chromophore in P7104R (B) and N2098R (C, CyR of *Calothrix* sp. NIES-2098, PDB code 6LM0). UV–Vis spectra of P7104R mutants (D), rearranged in putative critical amino acids contributing to color-tuning.

Spectroscopic differences between CyR and CyR-II were also investigated. The absorption maxima of dark-adapted purified P7104R, CBR35R, and MAG18R were located at 570, 565.5, and 546.5 nm, respectively (Fig. 3A). The present rhodopsin phylogeny revealed that the first and second belong to the sediment and soil group, and the third belongs to the marine bacterial mat and biofilm group. The two groups of CyR-II were therefore characterized by utilization of yellow (around 570 nm) or green lights (550 nm), respectively. The former can utilize longer wavelength light than the latter and CyRs. We thus named the sediment and soil group Yellow-absorbing CyR-II (YCyR-II) and the bacterial mats and biofilms group Green-absorbing CyR-II (GCyR-II).

We then performed detailed spectroscopic analyses using P7104R because it has the longest absorption maximum among the three CyR-IIs. Although the absorption maximum of light- and dark-adapted BR have been known to differ [16, 46], the absorption characteristic of P7104R did not vary between experiments with (light-adapted) and without (dark-adapted) illumination (Table 1 and Fig. S4A). The retinal configuration of P7104R was estimated that predominantly all-*trans* isomeric state of retinal (75.8% in dark-adapted and 88.0% in light-adapted) based on the areas of peaks in the HPLC patterns using absorption coefficients of retinal isomers (Fig. S4B). This differed from that of the haloarchaeal BR but resemble that of other bacterial rhodopsins, including cyanobacterial CyR (N2098R) and GR (Table 1).

**Table 1:** Photochemical properties of light-driven outward proton pump rhodopsins.

Opsin type	λ_max_ (nm)	Retinal configuration	M-decay rate constant	p*K*_a_ value	Refs
P7104R	570	All-*trans* (predominant)	~0.0009 ms^−1^	<1.1 and ~6.6 (Asp85) ~10.8 (Lys215)	This study
N2098R	550^a^	All-*trans* (predominant)^a^	~0.016 ms^−1a^	<2.0 (Asp74)^a^ ~10.7 (Lys204)^a^	[21]^a^
GR	544^b^	All-*trans* (predominant)^c,d^	~2.3 ms^−1c^, ~1 ms^−1e^	~5.9 (Asp121)^b^ ~9.12 (Lys257)^b^	[47]^b^, [10]^c^, [48]^d^, [49]^e^
BR	570^f^	All-*trans* (~50%)^g^ 13-*cis* (~50%)^g^	~0.25 ms^−1h^	~2.6 (Asp85)^i^ ~13.3 (Lys216)^j^	[16]^f^, [46]^g^, [50]^h^, [51]^i^, [52]^j^
PR (GPR)	520^k^	All-*trans* (predominant)^l^	~4 ms^−1l^	~7.9 (Asp97)^m^ ~11.3 (Lys227)^n^	[18]^k^, [53]^l^, [54]^m^, [55]^n^
PR (BPR)	490^o^	N.D.	N.D.	~6.2 and ~7.9 (Asp97)^p^ N.D. (Lys227)	[17]^o^, [56]^p^

The data presented, except for P7104R, refer previous studies corresponding to a–p.

The photochemical reactions of microbial rhodopsin are triggered by absorbing visible light and isomerization of the retinal chromophore from all-*trans* to the 13-*cis* form. Subsequent to the retinal isomerization, the rhodopsin protein forms various photo-intermediates (e.g. K, L, M, N, and O intermediates in BR) [14] and returns to its original state within a picosecond-to-second timeframe. During the photocycle, the microbial rhodopsins show their cognate protein functions such as ion transport [15]. Among the microbial rhodopsins, the H^+^ pumping rhodopsins vectorially transfer a proton from the intracellular to the extracellular side during a single photocycle step composed of various sequentially formed photo-intermediates [15]. Therefore, the lifetime of the intermediate is directly related to the H^+^ transport activity. The flash-photolysis analyses using the purified P7104R were thus performed to reveal its ion-transport mechanism. The absorption at around 570 nm, which was emerged from the absorption maximum of P7104R, was bleached but recovered within 10 000 ms (Fig. S4C). In addition, two positive peaks were observed around 415 and 675 nm: these peaks appeared within 0.1 and 0.01 ms, respectively, and they bleached within 10 000 ms. Based on the time and location of the absorption maxima, we postulated that the changes of absorbance at 415 and 675 nm were attributable to the K- and M-intermediates, respectively (Fig. S4D). The photocycle of P7104R was thus completed by about 8000 ms after completing the following reactions: the K- and M-intermediates were formed within a microsecond time frame; then, when the K-intermediate was bleached, the M-intermediate continued to be sustained; subsequently, the M-intermediate was bleached, and the rhodopsin protein returned to its original state (570 nm). This photocycle was also observed in the *E. coli* membrane expressing P7104R (Fig. S4E and F). To elucidate the timing of proton uptake and release during the photocycle of P7104R, the absorption changes in the presence or absence of pyranine, a pH-sensitive dye, were monitored using the purified P7104R. A decrease in the pyranine signal was observed within 10 000 ms (Fig. S4D, gray line), indicating that the solvent was acidified. Because the solvent alkalization occurred within a time interval shorter than the detection limit, no increase in the signal was observed. Furthermore, by fitting a single exponential equation to the signal, the M-decay rate constant at pH 7.0 was estimated to be 0.0009 ms^−1^. This result indicated that the M-intermediate of P7104R was much more long-lived than the M-intermediates of the other light-driven outward H^+^ pumping rhodopsins, including N2098R (Table 1).

During the H^+^ transport in rhodopsins, the protons (H^+^) are transferred through some charged residues such as Asp, Glu, and Lys inside the proteins. These charged residues hence are known to be important for H^+^ transport. In other words, the different properties of these charged residues also imply different modes of H^+^ transport. We performed pH titration experiments to estimate the p*K*_a_ values of essential charged residues in P7104R (i.e. Asp85^P7104R^ and Lys215^P7104R^) and compared with other rhodopsins. The pH titration data of Asp85^P7104R^ were fitted to the Henderson–Hasselbalch equation assuming two p*K*_a_ values. The p*K*_a_ values were estimated to be <1.0 and 6.6 (Table 1, Fig. S4G–I). These p*K*_a_ values are similar to those of BPR and GPR but not to those of other cyanobacterial rhodopsins. The p*K*_a_ of Lys215^P7104R^ was estimated to be 10.8 based on fitting titration data to the Henderson–Hasselbalch equation with a single p*K*_a_ (Table 1, Fig. S4J–L). And that p*K*_a_ of Lys215^P7104R^ was equivalent to the p*K*_a_ of other rhodopsins. These results suggest that CyRs and CyR-IIs function as H^+^ pumps, but that the specific mechanism of H^+^ transport has been modified during the evolution of rhodopsins.

### Structure and red-tuning mechanism of P7104R

We elucidated which structural changes in the process of rhodopsin evolution cause the differences in spectroscopic properties, especially the absorption maximum. The crystal structure of cell-free-synthesized P7104R was solved at 2.07 Å to clarify the red-shifted absorption mechanism of P7104R (Table S1). The P7104R monomer was composed of seven transmembrane helices (helices A–G) as well as other rhodopsins (Fig. S5A and B). The overall structure of P7104R was more like that of N2098R (PDB ID code 6LM0, root-mean-square deviation [RMSD] 0.766 Å) [21] than BR (PDB ID code 1C3W, RMSD 1.44 Å) [42], but the structure of the β-sheet distributed outside the cell membrane was more similar to that of BR (Fig. S5A and B). A detailed comparison between the structure of P7104R and N2098R or BR (Fig. S5) showed that a pentagonal cluster near the retinal of P7104R, which is known to be important for proton transport, consisted of three water molecules adjacent to the Lys215^P7104R^, Asp85^P7104R^, and Asp211^P7104R^ (corresponding to Lys216^BR^, Asp85^BR^, and Asp212^BR^). This was also like that of N2098R (Fig. S5C and D). The structure around the proton exit was also similar in arrangement, although Glu (Glu182^N2098R^ and Glu194^BR^) was replaced by Lys193^P7104R^ (Fig. S5E and F). The cyclohexane ring comprising the retinal chromophore of P7104R was more slanted than those of N2098R and BR (Figs 3B and C, S5C, D, and G–I). A comparison of the amino acids around the retinal showed that the Ile171^N2098R^ was rearranged to a Trp182^P7104R^ containing a larger functional group, and the distorted structure of the retinal was canceled by Trp182^P7104R^, through Phe181^P7104R^ and Glu144^P7104R^ (Fig. S3, orange bold). These three residues were also completely conserved in yellow-absorbing CBR35R but not in green-absorbing MAG18R (Fig. S3, orange bold). These results suggest that Trp182^P7104R^ affects the structure of the retinal and contributes to making the absorption maximum longer in P7104R and CBR35R.

To validate this hypothesis, we performed mutational analyses of P7104R. The F181W, W182I, and F181W/W182I mutants of P7104R absorbed at wavelengths of 565.0, 565.5, and 557.5 nm, respectively, which were blue-shifted compared with the wild type of P7104R (λ_max_ = 570 nm) (Fig. 3D). These results provide support for the hypothesis and indicate that Phe181^P7104R^ and Trp182^P7104R^ play a role in the red-shift of the absorption maximum in YCyR-IIs. In contrast, these results also suggest that a large red-shift has occurred in conjunction with alterations of other amino acid residues, not solely Phe181^P7104R^ and Trp182^P7104R^.

## Discussions

A survey of cyanobacterial rhodopsins in assembled genomes and metagenomic contigs was performed. Phylogenetic analysis of the identified rhodopsins led to the discovery of a new cyanobacteria-specific rhodopsin clade, CyR-II, containing predominantly metagenome-derived sequences (Fig. 1A). CyR-II is the third rhodopsin clade unique to cyanobacteria and, together with CyHRs and CyRs, forms CyipRs, phylogenetically distinct cyanobacteria-specific rhodopsins. Phylogenomic analysis revealed that CyipRs, which evolved within cyanobacteria, are widely distributed among different cyanobacterial lineages (Figs 1C and S2). The inconsistent topology between the phylogeny of cyanobacterial species and CyipRs suggests that CyipRs spread within cyanobacterial lineages through repeated horizontal gene transfer (HGT) events. As an example of CyR-IIs, *Chroococcidiopsidaceae* MAGs (lineage B) and *Nodosilinea nodulosa* PCC 7104 (lineage C-II) are phylogenomically distant and both possess YCyR-IIs, suggesting that YCyR-IIs were transferred via HGT (Figs 1B and S2). In addition, CyRs of *Phormidesmis priestleyi* BC 1401 (lineage D) and CyHRs of two MAGs (lineage C-II) appear to have been acquired by HGTs (Fig. S1). Other than CyipRs, HGTs are also important for rhodopsin radiation in cyanobacterial lineages. In XeRs, multiple HGTs within cyanobacteria should have occurred due to the mixed cyanobacterial lineages within the XeR clade (Figs S1 and S2). Cyanobacterial NaRs and XLRs appear to have been acquired from noncyanobacterial lineages (Figs S1 and S2) [21]. Almost all cyanobacteria are known to possess the *diox1* gene for the biosynthesis of retinal, a rhodopsin chromophore [21], suggesting that cyanobacteria only need to acquire a rhodopsin gene for rhodopsin-mediated photosynthesis to function. Rhodopsin-mediated photosystem is a simpler light-utilizing system and requires roughly half of the maintenance energy per molecule of chromophore than the chlorophyll-based photosystem [57]. Thus, the evidence of repeated HGT events of rhodopsin genes in cyanobacterial lineages proposes that rhodopsin acquisition plays an important role in cyanobacterial survival.

Cyanobacteria show different rates of rhodopsin possession according to their morphology, which was previously defined by five sections (Table S2) [31]. In particular, the morphology section I (unicellular forms) shows a low rate of rhodopsin possession compared with sections II–V (Table S2). Strains in sections II–V are multicellular or filamentous, which causes cell aggregation during growth, resulting in competition for light energy among cells due to self-shading [58]. In addition, filamentous organisms (sections III–V) often form bacterial mats and are exposed to light competition among phototrophs within the mat [59]. These factors suggest that light utilization by rhodopsin may help cyanobacteria to survive in an environment where they compete for light with other cells. Indeed, high rhodopsin possession rate lineages such as the C-II, C-III, and D lineages are dominated by filamentous organisms (section III) (Fig. S2). In contrast, rhodopsin-mediated photosynthesis may not be important for the survival of sparsely distributed cyanobacterial cells (section I). For example, rhodopsin genes have not been detected in cyanobacteria of the C-I lineage (Fig. S2), such as *Prochlorococcus* and marine *Synechococcus*, which are free-living unicellular oceanic organisms, whereas most marine bacteria living in the photic zone are known to possess rhodopsins for light energy harvesting [19].

Heterologous expression analyses showed that the CyR-IIs function as light-driven outward H^+^ pumps (Fig. 2B). H^+^ pumping rhodopsins in cyanobacteria have already been found (e.g. XLRs and CyRs) [21,47], whereas PRs, the most diverse and abundant H^+^ pumping rhodopsins in the ocean, have never been found in any cyanobacteria. This suggests that PR is a light-driven H^+^ pump specialized for marine heterotrophs living in oligotrophic environments [19]. The representative CyR-II, P7104R, does not exhibit the dark-adapted and light-adapted phenotypes (Fig. S4A). Furthermore, the retinal configuration of P7104R is predominantly all-*trans* retinal (Fig. S4B, Table 1). It is known that BR forms a highly dense structure on the native membrane (called the purple membrane, BR:lipids = 75:25) [60], whereas the amount of other H^+^ pumping rhodopsins, including CyR, in the native membrane is significantly less [18]. If BR has only all-*trans* retinal, it induces a large pH change on the cytoplasmic side, resulting in unfavorable effects on the archaeon itself. In contrast, the pH change induced by other H^+^ pumping rhodopsins is much less. Thus, the presence of dark–light adaptation in BR and the absence of it in other H^+^ pumping rhodopsins seem to be reasonable. Cyanobacteria possess complex photoreceptor systems [61,62]. All of the photoreceptor proteins that have been studied in detail, such as phytochrome, phycocyanin, and phycoerythrin, work to increase the efficiency of photosynthesis [63,64]. In contrast, the light energy received by the retinal of CyR or CyR-II would be consumed as energy to transport H^+^ out of the membrane, independent of the photosynthetic system. It is generally considered that the electrochemical gradient generated by H^+^-transporting rhodopsin is used for ATP synthesis, flagellar rotation, and so on [65,66]. In order to elucidate the physiological functions of CyR and CyR-II, it is necessary to clarify their subcellular localization in future studies.

Both CyR and CyR-II function as light-driven outward H^+^ pumps, but the physical and spectroscopic characteristics of the rhodopsin proteins are slightly different (Figs 2B and 3A, and Table 1). These differences may have arisen during the process of cyanobacterial adaptation to their habitat. P7104R, which is the representative CyR-II, has a high p*K*_a_ value of proton acceptor (Asp74^P7104R^, <1.1 and ~6.6) and a lack of proton-releasing group amino acids (corresponding to Glu194^BR^ and Glu204^BR^) (Figs 2A, S3, and S5E and F); these properties of P7104R were more similar to PR than CyR (Table 1). These features may reflect that *Nodosilinea nodulosa* PCC 7104 harboring P7104R is isolated from the ocean (Figs 1B and S2). The high p*K*_a_ value in PR (Asp97^GPR^, ~7.9; Asp97^BPR^, ~6.2 and ~7.9) [54,56], presumably suitable for the weak alkaline marine environments, is stabilized via a hydrogen bond between Asp97^PR^ (corresponding to Asp79^BPR^) and His75^PR^ (His57^BPR^) [67]. Through our structural analysis, the high p*K*_a_ value in P7104R may also be stabilized via a hydrogen bond between Ser96^P7104R^ and His42^P7104R^ (Fig. S5E and F). In contrast to CyR, the proton donor (corresponding to Asp96^BR^) in some CyR-II is replaced by Ser (Ser96^P7104R^) (Figs 2A, S3, and S5J and K). This suggests that a proton is supplied directly to the Schiff base Lys215^P7104R^ from the environment, rather than from a proton donor [32]. The observed changes in the amino acid residues of the proton donor are indicative of a process of adaptation to the environment, whereby rhodopsin not only underwent modification to adapt to cyanobacteria, but also underwent further modification to adapt to each environment.

The CyR-II clade found in this study is divided into two groups: GCyR-IIs with an absorption maximum at 550 nm and YCyR-IIs at 570 nm (Figs 1B and 3A). Mutational analyses showed that the red-shifted absorption maximum of P7104R is attributed to the presence of a larger functional group amino acids (Phe181^P7104R^ and Trp182^P7104R^) around the retinal (Fig. 3D), which are residues that differ from those reported in PR [68]. Such red shifts in the absorption wavelength of photoreceptors have also been reported in chlorophyll-based photosystems, such as chlorophyll *d* [1] and *f* [69,70]. The BR (λ_max_ = 570 nm) possessed by halophilic archaea is also known to utilize relatively long-wavelength region light [16,60]. This absorption range is suitable for hypersaline lakes because carotenoids produced by hyper-halotolerant organisms in the upper layer, such as the unicellular green alga *Dunaliella salina* [71], absorb blue-to-green light. In other words, the light-absorbing range of rhodopsin as well as chlorophyll is highly dependent on the light conditions of their habitat [2]. The YCyR-II genes were found mainly in cyanobacteria inhabiting certain terrestrial environments, such as sediment and soil (Fig. 1B), suggesting that red-shifted rhodopsin may harvest light and function more efficiently in their habitats. In addition, the presence of cyanobacteria with both CyR and CyR-II (two *Chroococcidiopsidaceae* MAGs, see Figs S1 and S2) may also be advantageous because their absorption maximum does not overlap, suggesting flexible adaptation to different light conditions.

## Conclusions

Our survey of cyanobacterial rhodopsins in assembled genomes and metagenomic contigs and phylogenetic analysis revealed a new cyanobacteria-specific rhodopsin clade: CyR-II. CyR-IIs function as light-driven outward H^+^ pumps and are subdivided into two subclades: YCyR-II (λ_max_ = 570 nm) and GCyR-II (λ_max_ = 550 nm). In addition, the present rhodopsin phylogeny revealed that YCyR-II was mainly identified from sediment and soil, and GCyR-II from marine bacterial mat and biofilm. This study introduces a new key player, “rhodopsin,” into the history of cyanobacterial radiation to diverse environments, which was previously thought to be driven solely by chlorophyll diversification. Cyanobacteria may have expanded into a wide range of environments not only by diversifying the photosynthetic photoreceptor pigments, but also by acquiring rhodopsins and adjusting the absorption wavelength of the rhodopsins to adapt to the light conditions of their habitat.

## Supplementary Material

CyR-II_Hasegawa_SI_20240909_wrae175

## Data Availability

The coordinates and structure factor for P7104R have been deposited in the Protein Data Bank, www.wwpdb.org (PDB ID code 8H79). The sequences of cyanobacterial metagenome-assembled genomes and contigs used in this study and Supporting Data S1–S8 are available at Figshare (https://doi.org/10.6084/m9.figshare.26232335).

## References

[1] Miyashita H , Ikemoto H, Kurano N et al. Chlorophyll *d* as a major pigment. *Nature* 1996;383:402–2. 10.1038/383402a0

[2] Sanfilippo JE , Garczarek L, Partensky F et al. Chromatic acclimation in cyanobacteria: a diverse and widespread process for optimizing photosynthesis. *Ann Rev Microbiol* 2019;73:407–33. 10.1146/annurev-micro-020518-115738 31500538

[3] Urbach E , Robertson DL, Chisholm SW. Multiple evolutionary origins of prochlorophytes within the cyanobacterial radiation. *Nature* 1992;355:267–70. 10.1038/355267a0 1731225

[4] Larkum AW , Scaramuzzi C, Cox GC et al. Light-harvesting chlorophyll c-like pigment in *Prochloron*. *Proc Natl Acad Sci USA* 1994;91:679–83. 10.1073/pnas.91.2.679 11607451 PMC43012

[5] Partensky F , Hess WR, Vaulot D. *Prochlorococcus*, a marine photosynthetic prokaryote of global significance. *Microbiol Mol Biol Rev* 1999;63:106–27. 10.1128/MMBR.63.1.106-127.1999 10066832 PMC98958

[6] Miyashita H , Ohkubo S, Sorimachi Y et al. Discovery of chlorophyll *d* in *Acaryochloris marina* and chlorophyll *f* in a unicellular cyanobacterium, strain KC1, isolated from Lake Biwa. *J Phys Chem Biophys* 2014;4:149. 10.4172/2161-0398.1000149

[7] Sanfilippo JE , Nguyen AA, Karty JA et al. Self-regulating genomic island encoding tandem regulators confers chromatic acclimation to marine *Synechococcus*. *Proc Natl Acad Sci USA* 2016;113:6077–82. 10.1073/pnas.1600625113 27152022 PMC4889380

[8] Six C , Thomas J-C, Garczarek L et al. Diversity and evolution of phycobilisomes in marine *Synechococcus* spp.: a comparative genomics study. *Genome Biol* 2007;8:R259. 10.1186/gb-2007-8-12-r259 18062815 PMC2246261

[9] Jung K-H , Trivedi VD, Spudich JL. Demonstration of a sensory rhodopsin in eubacteria. *Mol Microbiol* 2003;47:1513–22. 10.1046/j.1365-2958.2003.03395.x 12622809

[10] Miranda MRM , Choi AR, Shi L et al. The photocycle and proton translocation pathway in a cyanobacterial ion-pumping rhodopsin. *Biophys J* 2009;96:1471–81. 10.1016/j.bpj.2008.11.026 19217863 PMC2717234

[11] Hasemi T , Kikukawa T, Kamo N et al. Characterization of a cyanobacterial chloride-pumping rhodopsin and its conversion into a proton pump. *J Biol Chem* 2016;291:355–62. 10.1074/jbc.M115.688614 26578511 PMC4697170

[12] Niho A , Yoshizawa S, Tsukamoto T et al. Demonstration of a light-driven SO_4_^2−^ transporter and its spectroscopic characteristics. *J Am Chem Soc* 2017;139:4376–89. 10.1021/jacs.6b12139 28257611

[13] Rokitskaya TI , Alekseev AA, Tsybrov FM et al. Retinal-based anion pump from the cyanobacterium *Tolypothrix campylonemoides*. *Biochem Mosc* 2023;88:1571–9. 10.1134/S0006297923100127 38105025

[14] Ernst OP , Lodowski DT, Elstner M et al. Microbial and animal rhodopsins: structures, functions, and molecular mechanisms. *Chem Rev* 2014;114:126–63. 10.1021/cr4003769 24364740 PMC3979449

[15] Kojima K , Sudo Y. Convergent evolution of animal and microbial rhodopsins. *RSC Adv* 2023;13:5367–81. 10.1039/D2RA07073A 36793294 PMC9923458

[16] Lozier RH , Bogomolni RA, Stoeckenius W. Bacteriorhodopsin: a light-driven proton pump in *Halobacterium halobium*. *Biophys J* 1975;15:955–62. 10.1016/S0006-3495(75)85875-9 1182271 PMC1334761

[17] Béjà O , Spudich EN, Spudich JL et al. Proteorhodopsin phototrophy in the ocean. *Nature* 2001;411:786–9. 10.1038/35081051 11459054

[18] Béjà O , Aravind L, Koonin EV et al. Bacterial rhodopsin: evidence for a new type of phototrophy in the sea. *Science* 2000;289:1902–6. 10.1126/science.289.5486.1902 10988064

[19] Fuhrman JA , Schwalbach MS, Stingl U. Proteorhodopsins: an array of physiological roles? *Nat Rev Microbiol* 2008;6:488–94. 10.1038/nrmicro1893 18475306

[20] Pinhassi J , DeLong EF, Béjà O et al. Marine bacterial and archaeal ion-pumping Rhodopsins: genetic diversity, physiology, and ecology. *Microbiol Mol Biol Rev* 2016;80:929–54. 10.1128/MMBR.00003-16 27630250 PMC5116876

[21] Hasegawa M , Hosaka T, Kojima K et al. A unique clade of light-driven proton-pumping rhodopsins evolved in the cyanobacterial lineage. *Sci Rep* 2020;10:16752. 10.1038/s41598-020-73606-y 33028840 PMC7541481

[22] Nishimura Y , Yoshizawa S. The OceanDNA MAG catalog contains over 50,000 prokaryotic genomes originated from various marine environments. *Sci Data* 2022;9:305. 10.1038/s41597-022-01392-5 35715423 PMC9205870

[23] Al-Amoudi S , Razali R, Essack M et al. Metagenomics as a preliminary screen for antimicrobial bioprospecting. *Gene* 2016;594:248–58. 10.1016/j.gene.2016.09.021 27642121

[24] Babilonia J , Conesa A, Casaburi G et al. Comparative metagenomics provides insight into the ecosystem functioning of the Shark Bay stromatolites. *Western Australia Front Microbiol* 2018;9:1359. 10.3389/fmicb.2018.01359 29988640 PMC6027182

[25] Camargo AP , de Souza RSC, de Britto CP et al. Microbiomes of Velloziaceae from phosphorus-impoverished soils of the *Campos rupestres*, a biodiversity hotspot. *Sci Data* 2019;6:140. 10.1038/s41597-019-0141-3 31366912 PMC6668480

[26] Zhang W , Ding W, Li YX et al. Marine biofilms constitute a bank of hidden microbial diversity and functional potential. *Nat Commun* 2019;10:517. 10.1038/s41467-019-08463-z 30705275 PMC6355793

[27] Eddy SR . Accelerated profile HMM searches. *PLoS Comput Biol* 2011;7:e1002195. 10.1371/journal.pcbi.1002195 22039361 PMC3197634

[28] Nguyen LT , Schmidt HA, Von Haeseler A et al. IQ-TREE: a fast and effective stochastic algorithm for estimating maximum-likelihood phylogenies. *Mol Biol Evol* 2015;32:268–74. 10.1093/molbev/msu300 25371430 PMC4271533

[29] Hoang DT , Chernomor O, von Haeseler A et al. UFBoot2: improving the ultrafast bootstrap approximation. *Mol Biol Evol* 2018;35:518–22. 10.1093/molbev/msx281 29077904 PMC5850222

[30] Walter JM , Coutinho FH, Dutilh BE et al. Ecogenomics and taxonomy of cyanobacteria phylum. *Front Microbiol* 2017;8:2132. 10.3389/fmicb.2017.02132 29184540 PMC5694629

[31] Rippka RY , Deruelles J, Waterbury JB et al. Generic assignments, strain histories and properties of pure cultures of cyanobacteria. *Microbiology* 1979;111:1–61. 10.1099/00221287-111-1-1

[32] Sudo Y , Yoshizawa S. Functional and photochemical characterization of a light-driven proton pump from the Gammaproteobacterium *Pantoea vagans*. *Photochem Photobiol* 2016;92:420–7. 10.1111/php.12585 26970049

[33] Furuse M , Tamogami J, Hosaka T et al. Structural basis for the slow photocycle and late proton release in *Acetabularia* rhodopsin I from the marine plant *Acetabularia acetabulum*. *Acta Crystallogr D Biol Crystallogr* 2015;71:2203–16. 10.1107/S1399004715015722 26527138

[34] Shimono K , Goto M, Kikukawa T et al. Production of functional bacteriorhodopsin by an *Escherichia coli* cell-free protein synthesis system supplemented with steroid detergent and lipid. *Protein Sci* 2009;18:2160–71. 10.1002/pro.230 19746358 PMC2786979

[35] Katsura K , Matsuda T, Tomabechi Y et al. A reproducible and scalable procedure for preparing bacterial extracts for cell-free protein synthesis. *J Biochem (Tokyo)* 2017;162:357–69. 10.1093/jb/mvx039 28992119 PMC7109869

[36] Hosaka T , Yoshizawa S, Nakajima Y et al. Structural mechanism for light-driven transport by a new type of chloride ion pump, *Nonlabens marinus* rhodopsin-3. *J Biol Chem* 2016;291:17488–95. 10.1074/jbc.M116.728220 27365396 PMC5016146

[37] Hirata K , Kawano Y, Ueno G et al. Achievement of protein micro-crystallography at SPring-8 beamline BL32XU. *J Phys Conf Ser* 2013;425:012002. 10.1088/1742-6596/425/1/012002

[38] Hirata K , Yamashita K, Ueno G et al. *ZOO*: an automatic data-collection system for high-throughput structure analysis in protein microcrystallography. *Acta Crystallogr Sect Struct Biol* 2019;75:138–50. 10.1107/S2059798318017795 PMC6400253 30821703

[39] Yamashita K , Hirata K, Yamamoto M. *KAMO*: towards automated data processing for microcrystals. *Acta Crystallogr Sect Struct Biol* 2018;74:441–9. 10.1107/S2059798318004576 PMC5930351 29717715

[40] McCoy AJ , Grosse-Kunstleve RW, Adams PD et al. *Phaser* crystallographic software. *J Appl Crystallogr* 2007;40:658–74. 10.1107/S0021889807021206 19461840 PMC2483472

[41] Adams PD , Afonine PV, Bunkóczi G et al. *PHENIX*: a comprehensive python-based system for macromolecular structure solution. *Acta Crystallogr D Biol Crystallogr* 2010;66:213–21. 10.1107/S0907444909052925 20124702 PMC2815670

[42] Luecke H , Schobert B, Richter HT et al. Structure of bacteriorhodopsin at 1.55 Å resolution. *J Mol Biol* 1999;291:899–911. 10.1006/jmbi.1999.3027 10452895

[43] Emsley P , Cowtan K. *Coot*: model-building tools for molecular graphics. *Acta Crystallogr D Biol Crystallogr* 2004;60:2126–32. 10.1107/S0907444904019158 15572765

[44] Shih PM , Wu D, Latifi A et al. Improving the coverage of the cyanobacterial phylum using diversity-driven genome sequencing. *Proc Natl Acad Sci USA* 2013;110:1053–8. 10.1073/pnas.1217107110 23277585 PMC3549136

[45] Mongodin EF , Nelson KE, Daugherty S et al. The genome of *Salinibacter ruber*: convergence and gene exchange among hyperhalophilic bacteria and archaea. *Proc Natl Acad Sci USA* 2005;102:18147–52. 10.1073/pnas.0509073102 16330755 PMC1312414

[46] Stoeckenius W , Lozier RH, Bogomolni RA. Bacteriorhodopsin and the purple membrane of halobacteria. *Biochim Biophys Acta BBA - Rev Bioenerg* 1979;505:215–78. 10.1016/0304-4173(79)90006-5 35226

[47] Choi AR , Shi L, Brown LS et al. Cyanobacterial light-driven proton pump, *Gloeobacter* rhodopsin: complementarity between rhodopsin-based energy production and photosynthesis. *PLoS One* 2014;9:e110643. 10.1371/journal.pone.0110643 25347537 PMC4210194

[48] Hashimoto K , Choi AR, Furutani Y et al. Low-temperature FTIR study of *Gloeobacter* rhodopsin: presence of strongly hydrogen-bonded water and long-range structural protein perturbation upon retinal photoisomerization. *Biochemistry* 2010;49:3343–50. 10.1021/bi100184k 20230053

[49] Iizuka A , Kajimoto K, Fujisawa T et al. Functional importance of the oligomer formation of the cyanobacterial H^+^ pump *Gloeobacter* rhodopsin. *Sci Rep* 2019;9:10711. 10.1038/s41598-019-47178-5 31341208 PMC6656774

[50] Mukhopadhyay AK , Dracheva S, Bose S et al. Control of the integral membrane proton pump, bacteriorhodopsin, by purple membrane lipids of *Halobacterium halobium*. *Biochemistry* 1996;35:9245–52. 10.1021/bi960738m 8703930

[51] Subramaniam S , Marti T, Khorana HG. Protonation state of Asp (Glu)-85 regulates the purple-to-blue transition in bacteriorhodopsin mutants Arg-82 → Ala and Asp-85 → Glu: the blue form is inactive in proton translocation. *Proc Natl Acad Sci USA* 1990;87:1013–7. 10.1073/pnas.87.3.1013 1967832 PMC53400

[52] Druckmann S , Ottolenghi M, Pande A et al. Acid-base equilibrium of the Schiff base in bacteriorhodopsin. *Biochemistry* 1982;21:4953–9. 10.1021/bi00263a019 7138840

[53] Dioumaev AK , Brown LS, Shih J et al. Proton transfers in the photochemical reaction cycle of proteorhodopsin. *Biochemistry* 2002;41:5348–58. 10.1021/bi025563x 11969395

[54] Sharaabi Y , Brumfeld V, Sheves M. Binding of anions to proteorhodopsin affects the Asp97 p*K*_a_. *Biochemistry* 2010;49:4457–65. 10.1021/bi901746b 20405821

[55] Imasheva ES , Balashov SP, Wang JM et al. Selectivity of retinal photoisomerization in proteorhodopsin is controlled by aspartic acid 227. *Biochemistry* 2004;43:1648–55. 10.1021/bi0355894 14769042

[56] Wang W-W , Sineshchekov OA, Spudich EN et al. Spectroscopic and photochemical characterization of a deep ocean proteorhodopsin. *J Biol Chem* 2003;278:33985–91. 10.1074/jbc.M305716200 12821661

[57] Nishihara A , Tsukatani Y, Azai C et al. Illuminating the coevolution of photosynthesis and bacteria. *Proc Natl Acad Sci USA* 2024;121:e2322120121. 10.1073/pnas.2322120121 38875151 PMC11194577

[58] Prufert-Bebout L , Paerl HW, Lassen C. Growth, nitrogen fixation, and spectral attenuation in cultivated *Trichodesmium* species. *Appl Environ Microbiol* 1993;59:1367–75. 10.1128/aem.59.5.1367-1375.1993 16348931 PMC182091

[59] Stal LJ . Cyanobacterial Mats and Stromatolites. In: Whitton BA (ed), Ecology of Cyanobacteria II: Their Diversity in Space and Time. The Netherlands, Dordrecht: Springer, 2012, 65–125, 10.1007/978-94-007-3855-3_4.

[60] Oesterhelt D , Stoeckenius W. Rhodopsin-like protein from the purple membrane of *Halobacterium halobium*. *Nature New Biol* 1971;233:149–52. 10.1038/newbio233149a0 4940442

[61] Mullineaux CW . How do cyanobacteria sense and respond to light? *Mol Microbiol* 2001;41:965–71. 10.1046/j.1365-2958.2001.02569.x 11555279

[62] Wiltbank LB , Kehoe DM. Diverse light responses of cyanobacteria mediated by phytochrome superfamily photoreceptors. *Nat Rev Microbiol* 2019;17:37–50. 10.1038/s41579-018-0110-4 30410070

[63] Fujita Y , Hattori A. Photochemical interconversion between precursors of phycobilin chromoproteids in *Tolypothrix tenuis*. *Plant Cell Physiol* 1962;3:209–20.

[64] Yeh K-C , Wu S-H, Murphy JT et al. A cyanobacterial phytochrome two-component light sensory system. *Science* 1997;277:1505–8. 10.1126/science.277.5331.1505 9278513

[65] DeLong EF , Béjà O. The light-driven proton pump proteorhodopsin enhances bacterial survival during tough times. *PLoS Biol* 2010;8:e1000359. 10.1371/journal.pbio.1000359 20436957 PMC2860490

[66] Martinez A , Bradley AS, Waldbauer JR et al. Proteorhodopsin photosystem gene expression enables photophosphorylation in a heterologous host. *Proc Natl Acad Sci USA* 2007;104:5590–5. 10.1073/pnas.0611470104 17372221 PMC1838496

[67] Hempelmann F , Hölper S, Verhoefen MK et al. His75-Asp97 cluster in green proteorhodopsin. *J Am Chem Soc* 2011;133:4645–54. 10.1021/ja111116a 21366243

[68] Man D , Wang W, Sabehi G et al. Diversification and spectral tuning in marine proteorhodopsins. *EMBO J* 2003;22:1725–31. 10.1093/emboj/cdg183 12682005 PMC154475

[69] Chen M , Schliep M, Willows RD et al. A red-shifted chlorophyll. *Science* 2010;329:1318–9. 10.1126/science.1191127 20724585

[70] Chen M , Li Y, Birch D et al. A cyanobacterium that contains chlorophyll *f* - a red-absorbing photopigment. *FEBS Lett* 2012;586:3249–54. 10.1016/j.febslet.2012.06.045 22796191

[71] Oren A , Rodríguez-Valera F. The contribution of halophilic bacteria to the red coloration of saltern crystallizer ponds. *FEMS Microbiol Ecol* 2001;36:123–30. 10.1016/S0168-6496(01)00124-6 11451516

