# Supplementary material for: Cyanorhodopsin-II represents a yellow-absorbing proton-pumping rhodopsin clade within cyanobacteria
Source: ISME J. 2024 Nov 1;18(1):wrae175. doi: 10.1093/ismejo/wrae175 (PMC11528372; doi:10.1093/ismejo/wrae175)
Supplement: CyR-II_Hasegawa_SI_20240909_wrae175 [file cyr-ii_hasegawa_si_20240909_wrae175.docx]

**Supporting Information for**

Cyanorhodopsin-II represents a Yellow-Absorbing Proton-Pumping Rhodopsin clade within Cyanobacteria.

Masumi Hasegawa-Takano, Toshiaki Hosaka, Keiichi Kojima, Yosuke Nishimura, Marie Kurihara, Yu Nakajima, Yoshiko Ishizuka-Katsura, Tomomi Kimura-Someya, Mikako Shirouzu, Yuki Sudo, Susumu Yoshizawa

**Corresponding authors:** Masumi Hasegawa-Takano and Susumu Yoshizawa

**E-mail:** mhasegawa@jamstec.go.jp (M.H.-T.), yoshizawa@aori.u-tokyo.ac.jp (S.Y.)

This PDF file includes:

Supporting Materials and Methods p. 2–8

Figures S1–S5 p. 9–15

Tables S1–S2 p. 16–17

SI References p. 18–22

Supporting Data S1–S8 are available at Figshare (https://doi.org/10.6084/m9.figshare.26232335).

## Supporting Materials and Methods

### Collection of genome and rhodopsin sequences and their phylogeny

Cyanobacterial genome assemblies and their coding sequences were downloaded from the National Center for Biotechnology Information (NCBI) and the U.S. Department of Energy Joint Genome Institute (JGI) Integrated Microbial Genomes (IMG) database. We identified rhodopsin genes in these genomes, as described later. In addition, we explored cyanobacterial rhodopsins from metagenomes, using the OceanDNA MAG catalog [1], and metagenome assemblies derived from metagenomes published in previous studies [2–5]. Metagenomes were assembled as described in a previous study [1]. Briefly, read quality filtering was performed using Trimmomatic v0.35 [6] and quality controlled reads were assembled using MEGAHIT v1.1.4 [7]. Genes encoding rhodopsins were explored using two hidden Markov models (HMMs; Supporting Data 1 and 2) [8] constructed from large-scale sequence alignments of known rhodopsins and these homologs. One HMM (subACV; containing 3,173 sequences) was constructed mainly from archaeal/channel/viral rhodopsins, cyanobacterial halorhodopsins (CyHRs), and cyanorhodopsins (CyRs). The other (subB; containing 2,275 sequences) was mainly from bacterial rhodopsins such as proteorhodopsins (PRs) and xanthorhodopsin-like rhodopsins (XLRs). These rhodopsin sequences were collected from NCBI, the UniProt Archive (UniParc; https://www.uniprot.org/help/uniparc), and the Marine Microbial Eukaryotic Transcriptome Sequencing Project (MMETSP) [9]. The profile HMMs were generated from protein sequence alignments using MAFFT (v. 7.453) with the “--genafpair” and “--maxiterate 1000” options [10]. Rhodopsin genes were identified using hmmsearch (HMMER v3.3) with an e-value threshold (<1e-5) and a length threshold (≥174 amino acids; in terms of the length of an HMM included in a hit) [11]. In total, we organized a set of 179 cyanobacterial genomes (67 genomes containing rhodopsin genes) and 6 metagenomic contigs (all contigs containing rhodopsin genes), which were used for subsequent analysis (Supporting Data 3). The quality of the genomes was assessed using CheckM (v. 1.0.11) [12]. Taxonomic assignment of the cyanobacterial genomes was performed using GTDB-Tk (v. 1.3.0) and confirmed their cyanobacterial origin. For the metagenome-derived genomes and contigs (n=8), the taxonomic assignment of the rhodopsin-containing contigs was validated using the taxonomic filter function of MAGRE (https://github.com/yosuken/MAGRE) [1], which incorporates CAT (v. 5.0.3) [13], to determine whether the contigs were indeed derived from cyanobacterial genomes (Supporting Data 4). As six contigs were assigned to cyanobacteria, but two contigs (SRS1479369_N0008256 and SRS2826567_N0001366) were assigned to bacteria and no further taxonomic resolution was available, we further examined the taxonomic assignment result on a gene-by-gene basis. We considered that these contigs are also cyanobacterial because more than half of the taxonomically assigned genes of each contig are assigned to cyanobacteria (Supporting Data 4).

A rhodopsin phylogenetic tree was reconstructed as follows. Rhodopsin core domains (i.e. a region to which the rhodopsin HMMs were aligned) were extracted from the rhodopsin sequences and aligned using MAFFT (v. 7.453) with the options “--genafpair” and “--maxiterate 1000” [10]. The phylogenetic tree was reconstructed using the “LG+F+R6” model of IQ-TREE (v. 1.6.12) [14]. Branch supports were obtained by 1000 ultrafast bootstrap searches [15]. This model was selected as the best model by ModelFinder [16]. Branch supports were obtained by 1000 ultrafast bootstrap searches [15].

A cyanobacterial phylogeny was reconstructed as follows. A multiple sequence alignment of phylogenetic marker proteins (120 universally conserved single-copy genes of bacteria) was constructed using the “classify_wf” workflow of GTDB-Tk (v. 1.3.0) [17]. The phylogenomic tree was constructed using the “LG+F+R9” model of IQ-TREE (v. 1.6.12) [14]. Branch supports were obtained by 1000 ultrafast bootstrap searches [15]. This model was selected as the best model by ModelFinder [16]. Branch supports were obtained by 1000 ultrafast bootstrap searches [15]. Habitat and morphological information was collected manually: habitat information is based on “isolation source” of genome assembly in NCBI or the previously report [18] and morphological classifications is based on the previously report [19].

Reconstructed trees were visualized using Interactive Tree Of Life (v. 3) [20]. Raw data files of sequence alignments and phylogenies of rhodopsin and cyanobacterial genomes are available (Supporting Data 5–8).

### Construction of plasmids

The codon-optimized DNA fragments to *Escherichia coli* encoding the CyR-II genes (P7104R, CBR35R, and MAG18R) were chemically synthesized by Eurofins Genomics (Japan) and was inserted into the pET21a (+) plasmid vector (Novagen, Germany) using the NdeI and XhoI restriction enzyme sites. This cloning strategy resulted in encoding hexahistidine at the C-terminus.

Site-directed mutations (F181W, W182I, and F181W/W182I mutants of P7104R and W170F, I171W, and W170F/I171W mutants of N2098R) were introduced separately into the P7104R or N2098R gene subcloned into the pET21a (+) plasmid vector (Novagen, Germany).

### Light-induced ion transport measurements of CyR-IIs

The plasmids were transformed into *E. coli* strain C41 (DE3) cells (Lucigen, USA) and incubated at 37°C on a Luria–Bertani medium agar plate (NaCl 10 g L^−1^, Bacto Tryptone 10 g L^−1^, Bacto Yeast Extract 5 g L^−1^, and Agar 15 g L^−1^, pH 7) containing 100 µg mL^−1^ ampicillin. Before protein expression, transformants were grown at 37°C in 100 mL of 2× YT medium (NaCl 5 g L^−1^, Bacto Tryptone 16 g L^−1^, and Bacto Yeast Extract 10 g L^−1^, pH 7) with 100 µg mL^−1^ ampicillin in a one-liter flask until the absorbance at 660 nm reached 0.2–0.6. Protein expressions were then induced at 37°C for 3–4 h by adding 0.1 mM isopropyl β-D-1-thiogalactopyranoside (IPTG; Sigma-Aldrich, USA) and 10 µM all-*trans*-retinal (Sigma-Aldrich, USA). The rhodopsin-expressing cells were collected by centrifugation (4400×*g* for 3 min; MX-305, Tomy Seiko Co., Ltd., Japan), and washed three times in 100 mM NaCl, and then resuspended in 6 mL of 100 mM NaCl for measurements. The cell suspension was placed in the dark until the pH of the sample had stabilized and was then illuminated by using a 300-W xenon lamp (MAX-303, Asahi Spectra, Co., Ltd., Japan) with a green band-pass filter (520 ± 10 nm; MX0520, Asahi Spectra, Co., Ltd., Japan) for 3 min. The light power was adjusted to approximately 7 mW cm^−2^ by using an optical power meter (#3664, Hioki, Japan). Light-induced pH changes were monitored with a pH electrode (LAQUA F-72 pH meter, HORIBA, Ltd., Japan) in the presence or absence of a protonophore, 30 µM carbonyl cyanide *m*-chlorophenylhydrazone (CCCP; Sigma-Aldrich, USA), to confirm that the changes in pH were caused by proton transport. All measurements were performed at 4°C.

### Protein purification and spectroscopic analysis of P7104R

For protein purification, the P7104R-expressing *E. coli* C41 (DE3) (Lucigen, USA) cells were resuspended in 7 mL of buffer containing 50 mM Tris-HCl (pH 8.0) and 500 mM NaCl. The cells were then disrupted by sonication (Branson SFX 250 Digital Sonifier, Branson Ultrasonics, USA) on ice-cold water for 5 min. Crude membranes were obtained by ultracentrifugation at 4°C (106,800×*g* for 30 min; Optima XPN-90 Ultracentrifuge with a SW 32Ti rotor, Beckman Coulter, USA) and solubilized with 1.0% (w/v) *n*-dodecyl-β-d-maltoside (DDM, Dojindo Lab., Japan). The solubilized P7104R was collected by ultracentrifugation at 4°C (106,800×*g* for 30 min) and purified by Ni^2+^ affinity column chromatography (HisTrap FF Ni^2+^-NTA affinity chromatography column, Cytiva, Japan) eluted with 20–500 mM imidazole at room temperature (~25°C). The purified sample was concentrated, and its buffer was replaced with a new buffer (10 mM Tris-HCl [pH 7.0], 50 mM NaCl, 0.05% DDM) using an Amicon Ultra Filter (10,000 *M*_w_ cut-off; Millipore, USA) by centrifugation at 4°C (5000×*g* for 20 min; MX-305, Tomy Seiko Co.).

All UV-Vis spectra were measured using a UV-2450 spectrophotometer (Shimadzu, Japan) at room temperature (~25°C) under room light.

The retinal isomer composition was determined by using high-performance liquid chromatography (HPLC; SPD-20A UV-VIS detector and LC-20AT pump, Shimadzu, Japan) [21, 22]. The retinal in a sample was extracted with hexane as retinal oxime after denaturation by methanol (final concentration = 69 % (v/v)) with hydroxylamine (final concentration = 35 mM; Sigma-Aldrich, USA). The peaks were monitored at 360 nm, and the flow rate was 1.0 ml min^−1^. Before analysis, each sample was dark-adapted for seven days and then light-adapted by irradiation of 570 ± 10 nm light for 10 min by using a 300-W xenon lamp (MAX-303, Asahi Spectra, Japan). The light power was adjusted to ~10 mW cm^−2^ by using an optical power meter (#3664, Hioki, Japan). The previously reported absorption coefficients of retinal isomers (51,600; 54,600; 30,600; 39,300; 29,600; 35,000; 52,100; and 49,000 cm^−1^ M^−1^ for all-*trans* 15-*anti*, all-*trans* 15-*syn*, 9-*cis* 15-*anti*, 9-*cis* 15-*syn*, 11-*cis* 15-*anti*, 11-*cis* 15-*syn*, 13-*cis* 15-*anti*, and 13-*cis* 15-*syn* retinal oximes, respectively) were used in the present calculation [22–25]. All measurements were conducted at room temperature (~25°C) in the dark.

### pH-dependent spectral changes of P7104R

For the determination of acid dissociation constants (p*K*_a_), purified P7104R was suspended in seven-mix buffer (Tris, citric acid, MES, HEPES, MOPS, CHES, and CAPS; 10 mM each) containing 50 mM NaCl and 0.05% DDM; this buffer has the same buffer capacity over a wide pH range. The initial pH of the buffer was approximately 6.8. The pH was then adjusted to the desired value (1.11–11.17) by adding a small amount of 1N HCl or NaOH, and the absorption spectrum (250–750 nm) was measured for samples under the various pH conditions. All UV-Vis spectra were measured using a UV-2600 spectrophotometer (Shimadzu, Japan) at room temperature (~25°C) under room light. The absorption difference (ΔAbs) from pH 6.8 at specific wavelengths was plotted against pH. The p*K*_a_ value or values were then estimated by fitting the data to the Henderson–Hasselbalch equation with two (p*K*_a1_ and p*K*_a2_) or one p*K*_a_ as follows:

ΔAbs = $\frac{a}{1+{10}^{(pH-pKa1)}}$+ $\frac{b}{1+{10}^{(pH-pKa2)}}$ + *c*,

ΔAbs = $\frac{d}{1+{10}^{(pH-pKa)}}$+ *e*,

where *a, b,* and *d* represent the amplitudes of the changes of the absorption differences, respectively, and *c* and *e* are offsets. After the experiment, the reversibility of the pH-dependent spectral changes of the samples was checked to confirm that the protein was not denatured during the experiment.

### Time-resolved transient absorption spectroscopy of P7104R

For the flash-photolysis experiment, the purified P7104R was concentrated, and its buffer exchanged to a new buffer (10 mM Tris-HCl [pH 7.0], 50 mM NaCl, 0.05% DDM) adjusted to 0.5 optical density mL^−1^ at 570 nm by using an Amicon Ultra Filter (10,000 *M*_w_ cut-off; Millipore, USA) and centrifugation at 4°C (5000×*g* for 20 min; MX-305, Tomy Seiko Co.). In addition, *E. coli* membranes expressing P7104R were disrupted by sonication (UD-200, Tomy Seiko Co.) on ice-cold water. Crude membranes were obtained by ultracentrifugation at 4°C (134,200×g for 30 min; Micro ultracentrifuge CS100FNX with S50A rotor, Hitachi Koki Co., Ltd., Japan) and homogenized in buffer (10 mM Tris, 50 mM NaCl, pH 7.0). Time-resolved absorption spectra from 370 to 720 nm at 5-nm intervals in purified P7104R and from 390 to 720 nm at 10-nm intervals in *E. coli* membrane expressing P7104R were measured by using a computer-controlled flash-photolysis system equipped with a Nd:YAG laser (Surelite I-10, Continuum, USA) as an actinic light source. The wavelength of the actinic light pulse was tuned to 565 nm using an optical parametric oscillator (Surelite OPO plus, Continuum, USA). The other parameters were set as previously described [26]. The data at 565 and 570 nm in purified P7104R were excluded from the analysis to account for the scattering of the light pulses. Data taken before the flash were used as the baseline. At each wavelength, 20 traces in purified P7104R and 20 to 400 traces in *E. coli* membrane expressing P7104R were averaged to improve the signal-to-noise ratio. All measurements were conducted at 25°C using a thermostat.

To observe proton uptake and release during the photocycle, pyranine (final concentration = 100 µM; Tokyo Chemical Industry Co., Ltd., Japan), which is pH-sensitive fluorochrome and has often been used to monitor light-induced pH changes in various rhodopsins (e.g., [26]), was used as a pH indicator. The buffer of purified P7104R was exchanged to an unbuffered solution (50 mM NaCl, 0.05% DDM, pH 7.0). The pH changes of the bulk environment were monitored as the absorption changes of pyranine at 450 nm. These changes were equated to the difference of absorption changes between the samples with and without pyranine. A total of 500 traces were averaged to improve the signal-to-noise ratio. All measurements were conducted at 25°C.

### P7104R protein crystallization

A codon-optimized DNA fragment containing the P7104R gene was amplified by the polymerase chain reaction and sub-cloned into the pCR2.1–TOPO vector (Thermo Fisher Scientific Life Sciences, USA), for the expression of the P7104R protein with histidine tag for affinity purification and the cleavage site for tobacco etch virus (TEV) protease at its N-terminus [27].

The P7104R protein used for crystallization was synthesized by using an *E. coli* cell-free protein synthesis system according to previously reported protocols used for NM-R3 production [27–30]. After a cell-free synthesis reaction and solubilization with DDM (Anatrace, USA), the protein was affinity purified on Ni-NTA Superflow resin (Qiagen, Germany). The His-affinity tag was cleaved off with TEV protease. The cleaved tag and the His-tagged TEV protease were removed by passage through Ni-NTA Superflow resin, and the P7104R protein was recovered from the flow-through fraction. The protein solution was concentrated and applied to a Superdex 200 10/300 column (Cytiva, Japan). The peak fractions containing the protein were pooled and concentrated again.

P7104R (55.1 mg/mL) protein was crystallized by the *in meso* method. The purified protein solution and monoolein (40:60 w/w) were homogenized, and the mixture was placed on a glass well plate using a micro-dispenser as previously described [27, 31]. Crystals of P7104R were grown at 20°C in 0.1 M HEPES (pH 6.8), 400 mM Li-sulfate, and 46% polyethylene glycol 400.

### Data collection, structure determination, and refinement

Diffraction data for P7104R were collected at the BL32XU beamline of the SPring-8 synchrotron by using the multiple small-wedge scheme implemented in the ZOO system [32, 33]. A total of nine small-wedge datasets were collected at 30° using a 10 × 15-μm beam. Data processing was performed using the KAMO program [34], and data from eight isomorphous small crystals were merged together to generate the final 2.07 Å dataset [35–37]. The structure was solved by molecular replacement using the Phaser program [38] in the Phenix suite [39]. The search model was the BR [40], and the structure was manually rebuilt with the Coot program [41]. Data collection and refinement statistics are presented in Table S1.


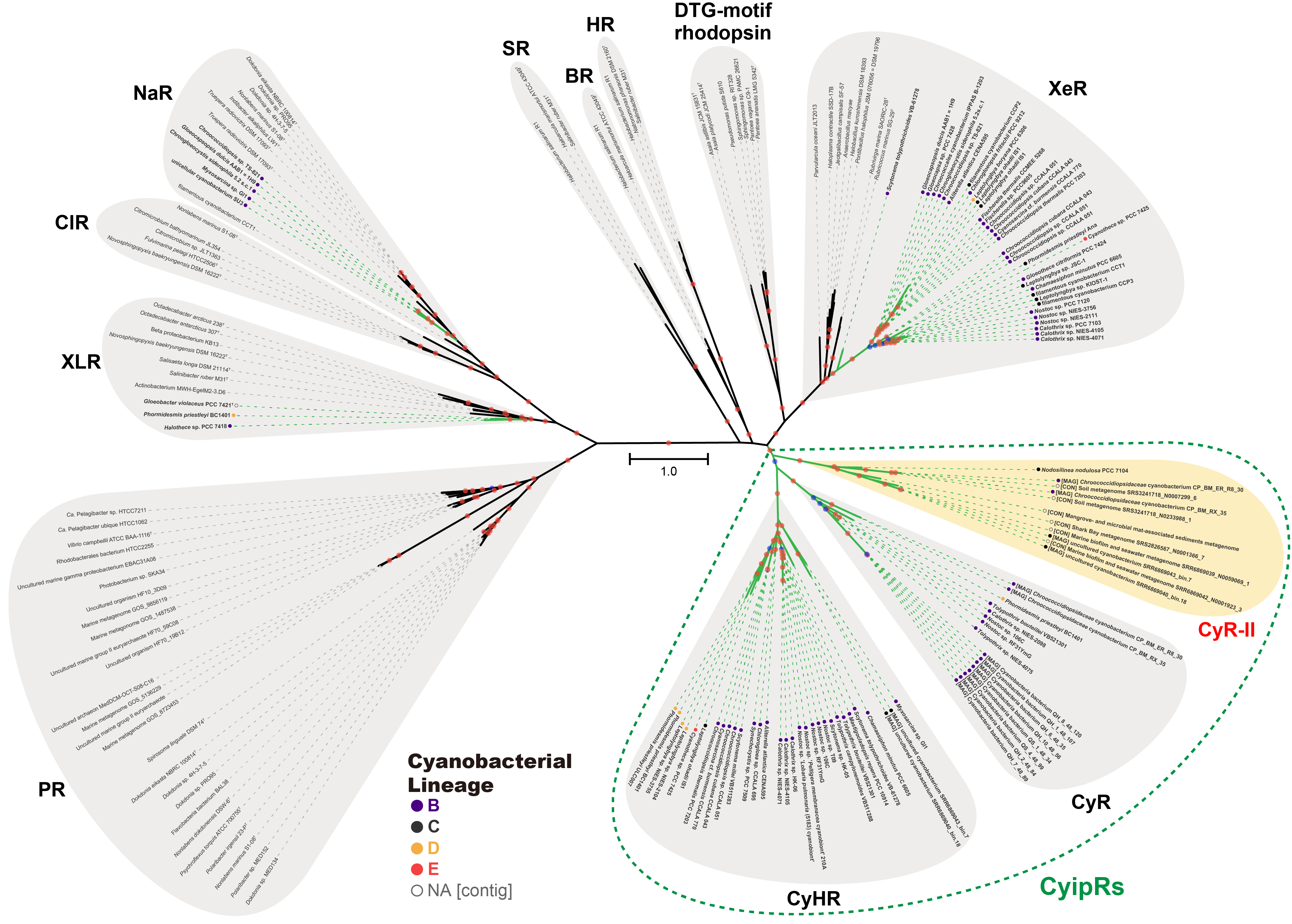


##### **Figure S1. Phylogenetic tree of prokaryotic rhodopsins.** A maximum likelihood tree of amino acid sequences of microbial rhodopsins. Phylogenetic analysis of the amino acid sequences was performed using the 102 cyanobacterial rhodopsin genes identified in this study and the 69 already-known rhodopsin genes. Bootstrap probabilities (≥50%) are indicated by colored circles. Green branches indicate cyanobacterial rhodopsins, and black branches indicate others. The circles in front of the sequence names were colored based on phylogenetic lineages that are referred to subclades in previous studies [42, 43], and strain names in thin gray are non-cyanobacterial strains. [CON] and [MAG] of cyanobacterial rhodopsins are indicated that the sequence is from metagenomic contig and MAG, respectively. Rhodopsin families are indicated as follows: NaR (Na^+^ pumping rhodopsin), ClR (Cl^–^ pumping rhodopsin), XLR (xanthorhodopsin-like rhodopsin), PR (proteorhodopsin), SR (sensory rhodopsin I and sensory rhodopsin II), BR (bacteriorhodopsin), HR (halorhodopsin), DTG-motif rhodopsin, XeR (xenorhodopsin), CyHR (cyanobacterial halorhodopsin), CyR (cyanorhodopsin), and an uncharacterized cyanobacteria-specific clade (cyanorhodopsin-II; CyR-II). The scale bar represents substitutions per site.


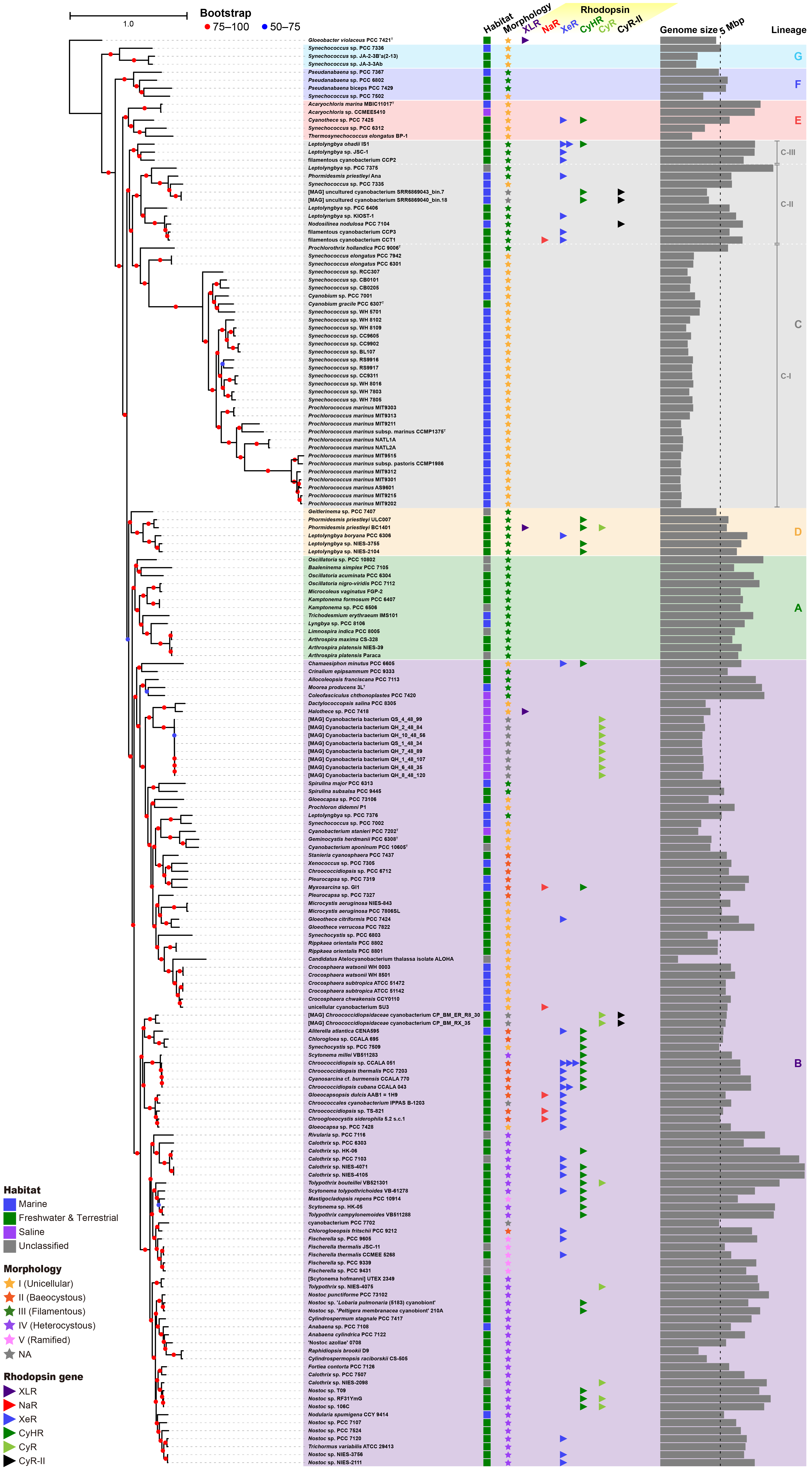


##### **Figure S2. Genome phylogenetic tree of cyanobacteria.** The tree was constructed by maximum likelihood estimation based on conserved phylogenetic marker proteins (120 ubiquitous single-copy proteins). Bootstrap probabilities (≥50%) are indicated by colored circles. Backgrounds were colored based on phylogenetic lineages. [CON] and [MAG] are indicated that the sequence is from metagenomic contig and MAG, respectively. Habitats and morphology of each strain are indicated by squares and stars in different colors, respectively. Habitat information is based on “isolation source” of genome assembly in NCBI or the previously report [18]. Morphological classifications are based on the previously report [19]. Presence of rhodopsin genes are indicated by right pointing triangles in different colors. The scale bar represents substitutions per site.


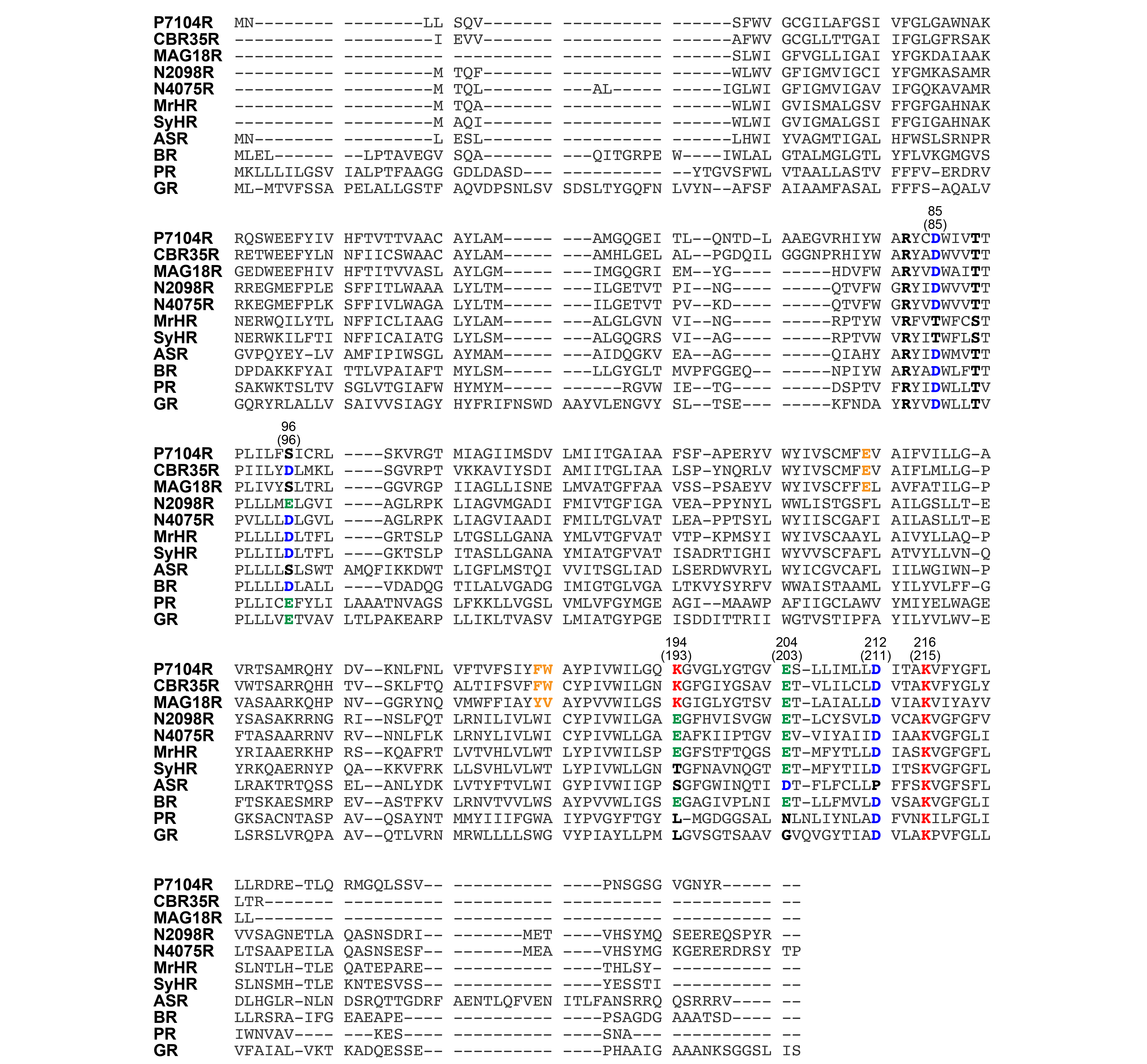


##### **Figure S3. Sequence alignment of rhodopsins.** Sequence alignment of rhodopsins. The accessions and rhodopsin families are as follows: P7104R (WP_017301364.1, CyR-II), CBR35R (MBV8882851.1, CyR-II), MAG18R (SRR6869040_N0001714_12, CyR-II), N2098R (BAY09002.1, CyR), N4075R (GAX43141.1, CyR), MrHR (WP_017314391.1, CyHR), SyHR (WP_009632765.1, CyHR), ASR (BAB74864.1, XeR), BR (CAP14056.1, BR), PR (AAG10475.1, PR), and GR (BAC88139.1, XLR). Columns of functionally important residues are shown in bold. The numbers above the columns indicate amino acid numbers in BR, and P7104R in parentheses. Known functions are as follows: primary proton acceptor (Asp85^BR^), proton donor (Glu96^BR^), proton release group (Glu194^BR^ and Glu204^BR^), counterion (Asp212^BR^), and Schiff base (Lys216^BR^). Two carboxylates, Asp (D) and Glu (E), are shown in blue and green, respectively, and Schiff base Lys (K) is shown in red. Amino acids in orange are involved in adjusting the absorption maxima of CyR-IIs.


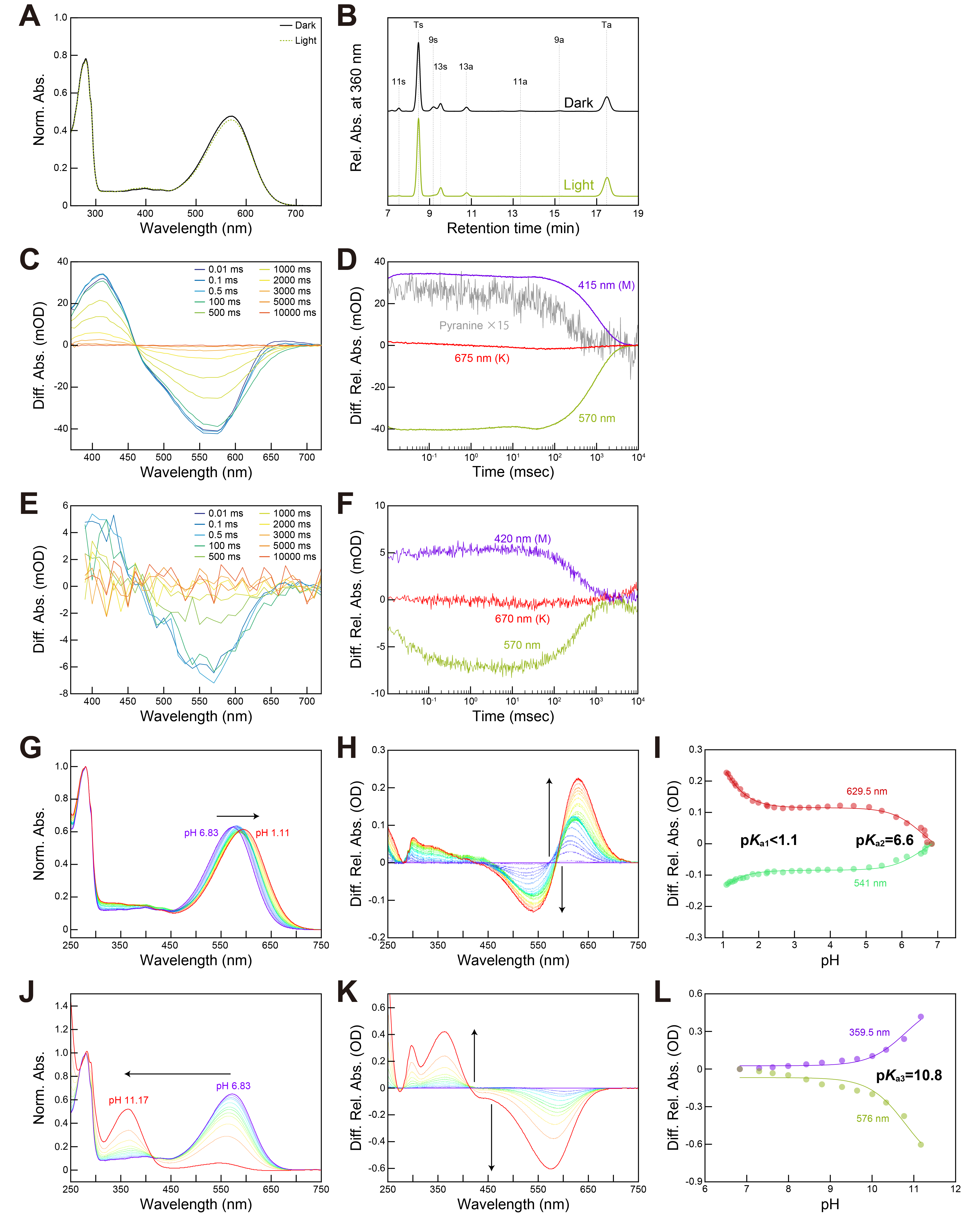


##### **Figure S4. Spectroscopic analyses of P7104R.** (**A**) UV-Vis spectra of P7104R with (yellow-green broken line) and without (black solid line) light illumination at 570 ± 10 nm for 10 min. (**B**) Retinal configuration of P7104R. HPLC patterns of retinal isomers of P7104R with (green line) and without (black line) light illumination at 570 ± 10 nm for 10 min. The retinal isomer exacted as retinal oximes. Molar comparison of each retinal isomer was calculated from areas of peaks in the HPLC patterns using absorption coefficients of retinal isomers (51,600; 54,600; 30,600; 39,300; 29,600; 35,000; 52,100; and 49,000 cm^−1^ M^−1^ for all-*trans* 15-*anti*, all-*trans* 15-*syn*, 9-*cis* 15-*anti*, 9-*cis* 15-*syn*, 11-*cis* 15-*anti*, 11-*cis* 15-*syn*, 13-*cis* 15-*anti*, and 13-*cis* 15-*syn* retinal oximes, respectively). The abbreviations of isomers are as follows: all-*trans* 15-*anti* (Ta), all-*trans* 15-*syn* (Ts), 9-*cis* 15-*anti* (9a), 9-*cis* 15-*syn* (9s),11-*cis* 15-*anti* (11a), 11-*cis* 15-*syn* (11s), 13-*cis* 15-*anti* (13a), and 13-*cis* 15-*syn* (13s) retinal oximes. (**C**) Flash-induced difference absorption spectra of purified P7104R over a spectral range of 370 to 720 nm and a time range of 0.01 to 10,000 ms. (**D**) Flash-induced kinetic data of purified P7104R at 415 nm (violet line, 20 traces), 570 nm (green line, 20 traces), and 675 nm (red line, 20 traces). The gray line represents the absorption changes of pyranine monitored at 450 nm (500 traces). (**E**) Flash-induced difference absorption spectra in *E. coli* membrane expressing P7104R over a spectral range of 390 to 720 nm and a time range of 0.01 to 10,000 ms. (**F**) Flash-induced kinetic data in *E. coli* membrane expressing P7104R at 420 nm (violet line, 2,000 traces), 570 nm (green line, 20 traces), and 670 nm (red line, 20 traces). (**G–L**) pH-induced spectral changes of N2098R over a spectral range of 250–750 nm. Sample was suspended in a seven mix buffer with 50 mM NaCl and 0.05% DDM. (**G**) Absorption spectra at acidic pH from 6.83 (violet line) to 1.11 (red line), where the pH was adjusted to the desired value by adding 1N HCl. (**H**) Difference spectra at acidic pH (6.83–1.11). Spectrum at pH 6.83 was subtracted from each spectrum and is described as a baseline. (**I**) Estimation of p*K*_a_ value of Asp85 in P7104R. Absorption differences at 514 nm (green circles) and 629.5 nm (red circles) were plotted against pH values. The data ranging from pH 1.11 to 6.83 were analyzed by using the Henderson-Hasselbalch equation with a single p*K*_a_ (solid lines). (**J**) Absorption spectra at alkaline pH from 6.833 (violet line) to 11.17 (red line), where the pH was adjusted to the desired value by adding 1N NaOH. (**K**) Difference spectra at alkaline pH (6.83–11.17). The spectrum at pH 6.83 was subtracted from each spectrum and is described as a baseline. (**L**) Estimation of the p*K*_a_ value of Lys215 in P7104R. Absorption differences at 359.5 nm (violet circles) and 576 nm (yellow-green circles) were plotted against pH values. The data ranging from pH 6.83 to 11.17 were analyzed by using the Henderson-Hasselbalch equation with a single p*K*_a_ (solid lines).


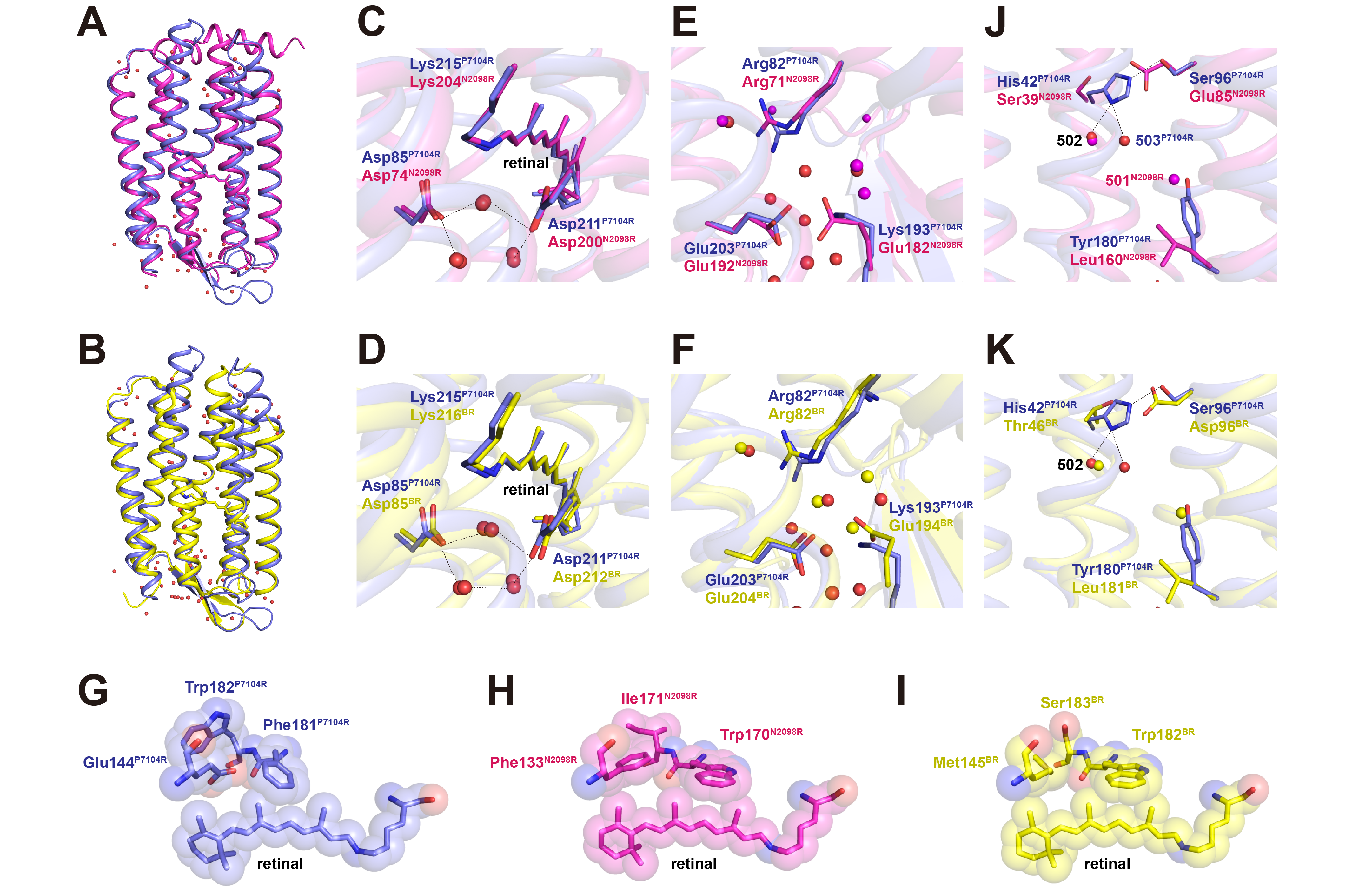


##### **Figure S5. Structural comparison between P7104R (violet; PDB code 8H79), N2098R (magenta; 6LM0), and BR (yellow; 1C3W).** Overall structural comparison between P7104R (violet) and N2098R (magenta) (**A**) or BR (**B**) viewed parallel to the membrane. Structural comparison of the retinal binding and water cluster region between P7104R (violet) and N2098R (magenta) (**C**) or BR (**D**). Structural comparison of the proton threshold region between P7104R (violet) and N2098R (magenta) (**E**) or BR (**F**). The amino acid positions around the retinal of P7104R (**G**), N2098R (**H**), and BR (**I**). Structural comparison of the proton release region between P7104R (violet) and N2098R (magenta) (**J**) or BR (**K**).

## Table S1. X-ray data collection, phasing and refinement statistics.

|  |  |  | P7104R |
| --- | --- | --- | --- |
| PDB ID | |  | 8H79 |
| *Data collection* | |  |  |
|  | Space group |  | *P32_1_* |
|  | Cell dimensions |  |  |
|  | a, b, c (Å) |  | 63.2, 63.2, 117.8 |
|  | α, β, γ (˚) |  | 90.0, 90.0, 120.0 |
|  | Wavelength (Å) |  | 1.0 |
|  | Resolution (Å) |  | 40.07 - 2.07  (2.144 - 2.07) |
|  | Total reflections |  | 213267 (21007) |
|  | Unique reflections |  | 17188 (1699) |
|  | Redundancy |  | 12.4 (12.4) |
|  | Completeness (%) |  | 99.8 (100) |
|  | *I* /σ(*I*) |  | 10.3 (1.6) |
|  | *R-merge* (%) |  | 22.7 (179.6) |
|  | *R-meas* (%) |  | 23.6 (187.4) |
|  | *CC_1/2_* (%) |  | 99.7 (53.5) |
| *Refinement* | |  |  |
|  | *R*_work_ (%) |  | 20.3 |
|  | *R*_free_ (%) |  | 24.8 |
|  | r.m.s.d. bond lengths (Å) |  | 0.005 |
|  | r.m.s.d. bond angles (˚) |  | 0.91 |
| Number of water molecules | |  | 46 |
| Average B factor (Å^2^) | |  |  |
|  | all |  | 29.6 |
|  | protein |  | 29.0 |
|  | ligand |  | 36.5 |
|  | solvent |  | 35.2 |
| *Ramachandran plot* | |  |  |
|  | Most favored regions (%) |  | 98.3 |
|  | outliers (%) |  | 0 |

Statistics for the highest-resolution shell are shown in parentheses.

**Table S2. Rhodopsin distributions in cyanobacteria with morphological information.**

| Morphology  (Section; Number of genomes) | Rhodopsin | | | | | | | | Possessing  rate ^†^ |
| --- | --- | --- | --- | --- | --- | --- | --- | --- | --- |
|  | XLR | NaR | XeR | CyHR | CyR | CyR-II | Total | Genome |  |
| I (68) | 2 | 1 | 4 | 3 | 0 | 0 | 10 | 8 | 12% |
| II (16) | 0 | 4 | 12 | 7 | 0 | 0 | 23 | 11 | 69% |
| III (40) | 1 | 1 | 9 | 5 | 1 | 1 | 18 | 13 | 33% |
| IV (35) | 0 | 0 | 7 | 13 | 5 | 0 | 25 | 19 | 54% |
| V (6) | 0 | 0 | 2 | 1 | 0 | 0 | 3 | 3 | 50% |
| NA ^*^ (14) | 0 | 0 | 1 | 2 | 10 | 4 | 17 | 13 | 93% |
| Metagenome contigs | 0 | 0 | 0 | 0 | 0 | 6 | 6 | – | – |
| Total | 3 | 6 | 35 | 31 | 16 | 11 | 102 | 67 | – |

^*^NA = “not available”

^†^Possessing rate = “Number of rhodopsin-harboring genomes”/“Number of genomes”

## SI References

#### 1. Nishimura Y, Yoshizawa S. The OceanDNA MAG catalog contains over 50,000 prokaryotic genomes originated from various marine environments. *Sci Data* 2022; **9**: 305.

#### 2. Al-Amoudi S, Razali R, Essack M, Amini MS, Bougouffa S, Archer JAC, et al. Metagenomics as a preliminary screen for antimicrobial bioprospecting. *Gene* 2016; **594**: 248–258.

#### 3. Babilonia J, Conesa A, Casaburi G, Pereira C, Louyakis AS, Reid RP, et al. Comparative metagenomics provides insight into the ecosystem functioning of the Shark Bay Stromatolites, Western Australia. *Front Microbiol* 2018; **9**: 1359.

#### 4. Camargo AP, de Souza RSC, de Britto Costa P, Gerhardt IR, Dante RA, Teodoro GS, et al. Microbiomes of Velloziaceae from phosphorus-impoverished soils of the *campos rupestres*, a biodiversity hotspot. *Sci Data* 2019; **6**: 140.

#### 5. Zhang W, Ding W, Li YX, Tam C, Bougouffa S, Wang R, et al. Marine biofilms constitute a bank of hidden microbial diversity and functional potential. *Nat Commun* 2019; **10**: 517.

#### 6. Bolger AM, Lohse M, Usadel B. Trimmomatic: a flexible trimmer for Illumina sequence data. *Bioinformatics* 2014; **30**: 2114–2120.

#### 7. Li D, Liu C-M, Luo R, Sadakane K, Lam T-W. MEGAHIT: an ultra-fast single-node solution for large and complex metagenomics assembly via succinct *de Bruijn* graph. *Bioinformatics* 2015; **31**: 1674–1676.

#### 8. Eddy SR. Accelerated profile HMM searches. *PLoS Comput Biol* 2011; **7**: e1002195.

#### 9. Keeling PJ, Burki F, Wilcox HM, Allam B, Allen EE, Amaral-Zettler LA, et al. The marine microbial eukaryote transcriptome sequencing project (MMETSP): Illuminating the functional diversity of eukaryotic life in the oceans through transcriptome sequencing. *PLoS Biol* 2014; **12**: e1001889.

#### 10. Katoh K, Standley DM. MAFFT multiple sequence alignment software version 7: Improvements in performance and usability. *Mol Biol Evol* 2013; **30**: 772–780.

#### 11. Potter SC, Luciani A, Eddy SR, Park Y, Lopez R, Finn RD. HMMER web server: 2018 update. *Nucleic Acids Res* 2018; **46**: W200–W204.

#### 12. Parks DH, Imelfort M, Skennerton CT, Hugenholtz P, Tyson GW. CheckM: assessing the quality of microbial genomes recovered from isolates, single cells, and metagenomes. *Genome Res* 2015; **25**: 1043–1055.

#### 13. von Meijenfeldt FAB, Arkhipova K, Cambuy DD, Coutinho FH, Dutilh BE. Robust taxonomic classification of uncharted microbial sequences and bins with CAT and BAT. *Genome Biol* 2019; **20**: 217.

#### 14. Nguyen LT, Schmidt HA, Von Haeseler A, Minh BQ. IQ-TREE: A fast and effective stochastic algorithm for estimating maximum-likelihood phylogenies. *Mol Biol Evol* 2015; **32**: 268–274.

#### 15. Hoang DT, Chernomor O, Von Haeseler A, Minh BQ, Vinh LS. UFBoot2: Improving the ultrafast bootstrap approximation. *Mol Biol Evol* 2018; **35**: 518–522.

#### 16. Kalyaanamoorthy S, Minh BQ, Wong TKF, Von Haeseler A, Jermiin LS. ModelFinder: Fast model selection for accurate phylogenetic estimates. *Nat Methods* 2017; **14**: 587–589.

#### 17. Parks DH, Chuvochina M, Waite DW, Rinke C, Skarshewski A, Chaumeil PA, et al. A standardized bacterial taxonomy based on genome phylogeny substantially revises the tree of life. *Nat Biotechnol* 2018; **36**: 996–1004.

#### 18. Walter JM, Coutinho FH, Dutilh BE, Swings J, Thompson FL, Thompson CC. Ecogenomics and taxonomy of cyanobacteria phylum. *Front Microbiol* 2017; **8**: 2132.

#### 19. Rippka RY, Deruelles J, Waterbury JB, Herdman M, Steiner RY. Generic assignments, strain histories and properties of pure cultures of cyanobacteria. *Microbiology* 1979; **111**: 1–61.

#### 20. Letunic I, Bork P. Interactive tree of life (iTOL) v3: an online tool for the display and annotation of phylogenetic and other trees. *Nucleic Acids Res* 2016; **44**: W242–W245.

#### 21. Sudo Y, Yoshizawa S. Functional and photochemical characterization of a light-driven proton pump from the Gammaproteobacterium *Pantoea vagans*. *Photochem Photobiol* 2016; **92**: 420–427.

#### 22. Groenendljk GWT, De Grip WJ, Daemen FJM. Quantitative determination of retinals with complete retention of their geometric configuration. *Biochim Biophys Acta BBALipids Lipid Metab* 1980; **617**: 430–438.

#### 23. Groenendijk GWT, De Grip WJ, Daemen FJM. Identification and characterization of *syn*- and *anti*-isomers of retinaloximes. *Anal Biochem* 1979; **99**: 304–310.

#### 24. Trehan A, Liu RSH, Shichida Y, Imamoto Y, Nakamura K, Yoshizawa T. On retention of chromophore configuration of rhodopsin isomers derived from three dicis retinal isomers. *Bioorganic Chem* 1990; **18**: 30–40.

#### 25. Matsuyama T, Yamashita T, Imamoto Y, Shichida Y. Photochemical properties of mammalian melanopsin. *Biochemistry* 2012; **51**: 5454–5462.

#### 26. Inoue S, Yoshizawa S, Nakajima Y, Kojima K, Tsukamoto T, Kikukawa T, et al. Spectroscopic characteristics of *Rubricoccus marinus* xenorhodopsin (*Rm*XeR) and a putative model for its inward H^+^ transport mechanism. *Phys Chem Chem Phys* 2018; **20**: 3172–3183.

#### 27. Hosaka T, Yoshizawa S, Nakajima Y, Ohsawa N, Hato M, DeLong EF, et al. Structural mechanism for light-driven transport by a new type of chloride ion pump, *Nonlabens marinus* rhodopsin-3. *J Biol Chem* 2016; **291**: 17488–17495.

#### 28. Furuse M, Tamogami J, Hosaka T, Kikukawa T, Shinya N, Hato M, et al. Structural basis for the slow photocycle and late proton release in *Acetabularia* rhodopsin I from the marine plant *Acetabularia acetabulum*. *Acta Crystallogr D Biol Crystallogr* 2015; **71**: 2203–2216.

#### 29. Shimono K, Goto M, Kikukawa T, Miyauchi S, Shirouzu M, Kamo N, et al. Production of functional bacteriorhodopsin by an *Escherichia coli* cell-free protein synthesis system supplemented with steroid detergent and lipid. *Protein Sci* 2009; **18**: 2160–2171.

#### 30. Katsura K, Matsuda T, Tomabechi Y, Yonemochi M, Hanada K, Ohsawa N, et al. A reproducible and scalable procedure for preparing bacterial extracts for cell-free protein synthesis. *J Biochem (Tokyo)* 2017; **162**: 357–369.

#### 31. Hato M, Hosaka T, Tanabe H, Kitsunai T, Yokoyama S. A new manual dispensing system for in meso membrane protein crystallization with using a stepping motor-based dispenser. *J Struct Funct Genomics* 2014; **15**: 165–171.

#### 32. Hirata K, Kawano Y, Ueno G, Hashimoto K, Murakami H, Hasegawa K, et al. Achievement of protein micro-crystallography at SPring-8 beamline BL32XU. *J Phys Conf Ser* 2013; **425**: 012002.

#### 33. Hirata K, Yamashita K, Ueno G, Kawano Y, Hasegawa K, Kumasaka T, et al. *ZOO*: an automatic data-collection system for high-throughput structure analysis in protein microcrystallography. *Acta Crystallogr Sect Struct Biol* 2019; **75**: 138–150.

#### 34. Yamashita K, Hirata K, Yamamoto M. *KAMO*: towards automated data processing for microcrystals. *Acta Crystallogr Sect Struct Biol* 2018; **74**: 441–449.

#### 35. Kabsch W. *XDS*. *Acta Crystallogr D Biol Crystallogr* 2010; **66**: 125–132.

#### 36. Evans G, Axford D, Waterman D, Owen RL. Macromolecular microcrystallography. *Crystallogr Rev* 2011; **17**: 105–142.

#### 37. Foadi J, Aller P, Alguel Y, Cameron A, Axford D, Owen RL, et al. Clustering procedures for the optimal selection of data sets from multiple crystals in macromolecular crystallography. *Acta Crystallogr D Biol Crystallogr* 2013; **69**: 1617–1632.

#### 38. McCoy AJ, Grosse-Kunstleve RW, Adams PD, Winn MD, Storoni LC, Read RJ. *Phaser* crystallographic software. *J Appl Crystallogr* 2007; **40**: 658–674.

#### 39. Adams PD, Afonine P V., Bunkóczi G, Chen VB, Davis IW, Echols N, et al. *PHENIX*: a comprehensive python-based system for macromolecular structure solution. *Acta Crystallogr D Biol Crystallogr* 2010; **66**: 213–221.

#### 40. Luecke H, Schobert B, Richter HT, Cartailler JP, Lanyi JK. Structure of bacteriorhodopsin at 1.55 Å resolution. *J Mol Biol* 1999; **291**: 899–911.

#### 41. Emsley P, Cowtan K. *Coot*: Model-building tools for molecular graphics. *Acta Crystallogr D Biol Crystallogr* 2004; **60**: 2126–2132.

#### 42. Shih PM, Wu D, Latifi A, Axen SD, Fewer DP, Talla E, et al. Improving the coverage of the cyanobacterial phylum using diversity-driven genome sequencing. *Proc Natl Acad Sci U S A* 2013; **110**: 1053–1058.

#### 43. Hasegawa M, Hosaka T, Kojima K, Nishimura Y, Nakajima Y, Kimura-Someya T, et al. A unique clade of light-driven proton-pumping rhodopsins evolved in the cyanobacterial lineage. *Sci Rep* 2020; **10**: 16752.
